# Slowing Down Water: Enhanced and Cation‐responsive MRI Contrast

**DOI:** 10.1002/anie.5723493

**Published:** 2026-05-20

**Authors:** Connor M. Ellis, James P. Smith, Matthew F. Allen, Ferenc E. Mózes, Stephen Faulkner, Jason J. Davis

**Affiliations:** ^1^ Department of Chemistry University of Oxford Oxford UK; ^2^ Oxford Centre for Clinical Magnetic Resonance Research Radcliffe Department of Medicine University of Oxford, Level 0, John Radcliffe Hospital Oxford UK

**Keywords:** Contrast agent, mesoporous silica nanoparticles, MRI, outer‐sphere, potassium‐responsive

## Abstract

The confinement of paramagnetic centres to the internal channels of mesoporous nanoparticles brings with it a much greater contribution from outer sphere effects by virtue of tuneable side wall association and a resulting much stretched water diffusion coefficient. We specifically utilise the relative ease of side wall modifications to demonstrate that kosmotropic (water‐ordering) effects can be leveraged to greatly increase the viscosity of water in the vicinity of internalised paramagnetic complexes. This has the effect of supporting unprecedented levels of image contrast with *q* = 0 systems and much enhanced *T*_1_ contrast for common *q* = 1 complexes, at clinically relevant field strengths. Physiologically relevant potassium‐responsive imaging was subsequently enabled by modulating the water organisation initially imparted by internalised crown ethers on specific cation binding.

## Graphical abstract

The controlled modification of mesoporous silica nanoparticle side walls with kosmotropic motifs supports an ability to reduce water diffusive mobility and dramatically increase MRI contrast to levels unprecedented for highly kinetically stable Gd‐DOTA configurations (both q = 1 and q = 0). These OS effects can further enable cation‐selective contrast with crown ether side wall modifications.

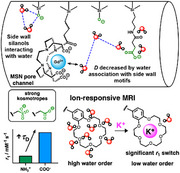

## 1 Introduction

Magnetic Resonance Imaging (MRI) is one of the most powerful non‐invasive diagnostic tools available to clinicians [1, 2, 3]. Exogenous contrast agents (CAs) have expanded the applications of MRI, not only for anatomical studies, but also in providing valuable information on tissue physiology. Currently, clinical CAs are typically gadolinium‐based contrast agents (GBCAs), such as gadoteric acid (Gd‐DOTA) [4, 5, 6]. They are classified as *T*_1_ agents, meaning they generate positive contrast by shortening the longitudinal relaxation times of water protons in vivo, and are often clinically employed to improve diagnostic utility [7, 8]. However, these molecular chelates present low longitudinal relaxivity (*r*_1_) values at typically used clinical fields (i.e., 2.9 mM^−1^ s^−1^ at 1.5 T, recorded in water at 37°C, for Gd‐DOTA) [4, 5, 6]. Consequently, a high dose; 0.1–0.3 mmol kg^−1^, is required to achieve sufficient contrast‐to‐noise (CNR) [9]. A range of strategies have been developed to enhance intrinsic CA relaxivity, including the confinement of Gd^3+^‐ion clusters within carbon nanotubes [10]. This approach demonstrated unprecedented relaxivities, albeit with Solomon‐Bloembergen‐Morgan (SBM) analyses that were unable to fully account for the resolved magnitude of *r*_1_. Additionally, the architecture lacked chemical tuneability, a powerful asset in the design of stimuli‐responsive agents. The immobilisation of GBCAs onto or within nanoparticle scaffolds, especially withinin the (ca. 3 nm in diameter) pore channels of mesoporous silica nanoparticles (MSNs), presents a powerful and saliently tuneable means of amplifying signal [8]. Furthermore, appropriately doped MSNs are able to support dramatically improved MRI scan sensitivity with good biocompatibility [11, 12]. It is worth noting that both Gd^3+^ locality and local water mobility within these nanoparticulate constructs are important in dramatically augmenting developed contrast (see below) [13, 14, 15].

The *r*_1_ of a MRI CA is governed by its constituent inner‐sphere (IS), second‐sphere (SS) and outer‐sphere (OS) contributions. OS effects arise from the translational diffusion of water molecules near the paramagnetic ion and are strongly influenced by the associated diffusion correlation time (*τ*_D_ ∝ 1/*D*) [16, 17, 18]. Whilst traditionally overlooked, these may constitute a very substantial (up to 40%) contribution to the observed *r*_1_ even for molecular chelates [19, 20].

**FIGURE 1:**
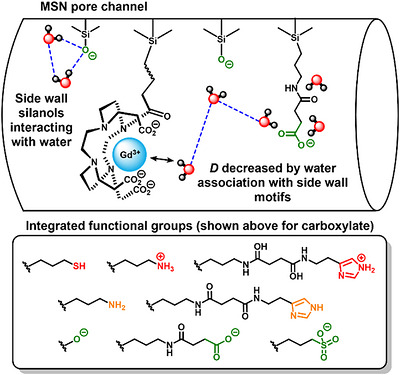
A schematic illustrating the different side wall chemical modifications and their associated influence on internalised water viscosity. Significant water ordering is expected when the side wall motif is kosmotropic (native silanol, carboxylated, sulfonated); restricting diffusive water mobility by up to two orders of magnitude, significantly increasing the associated CA *r*_1_.

The properties of nanoconfined water differ to those of the bulk by virtue of the significant interfacial interactions [21], leading, in some cases, to a high degree of local order and a reduced mobility that enhances *r*_1_ [22, 23, 24]. In particular, the silanol termini of silica surfaces have been reported to engage in a particularly strong hydrogen‐bonding based association with water [25, 26, 27, 28, 29]. Recent studies have demonstrated that these interactions, and increased surface polarity, dramatically reduce water mobility [30, 31, 32, 33, 34] with diffusion coefficients that are very notably lower than bulk (∼10^−10^–10^−11^ m^2^ s^−1^) [35]. Given this background, we hypothesised that a considered manipulation of this local water mobility could have a profound influence on relaxivity and so supported image contrast. We specifically considered that the introduction of additional water‐ordering (i.e., kosmotropic) and ion‐binding supramolecular receptive units could pave the way towards contrast agents responsive to physiologically relevant stimuli. To this end, we have systematically investigated the role of integrated side wall kosmotropes on nanoparticle generated proton relaxivity (Figure 1) [36]. We demonstrate that the microenvironment generated by these functional groups enables the relaxometric behaviour of GBCA‐doped MSNs to be tuned, and relaxivity levels achieved, to be unprecedented. This in itself can be leveraged in generating a library of potent, high signal:noise, CAs, including, as demonstrated below, with highly kinetically stable *q* = 0 chelates (generally capable of supporting only very modest contrast) [37, 38, 39]. While the architecture included in this work focuses on MSN CAs, one would fully expect this approach to be equally applicable to other nanoparticulate agents (e.g., *T*_1_‐active superparamagnetic iron oxide nanoparticles) where OS effects are often similarly overlooked [40].

An ability to spatially resolve cation presence has significant value in supporting our understanding and diagnosis of cancer and neurological conditions; K^+^ itself is implicit in maintaining electrolyte balance and resting membrane potential, disruption of which is indicative of the onset of a number of diseases (e.g., neurological or cardiac) [41, 42]. A direct imaging of K^+^ imbalance would enable an early diagnosis, providing valuable insight into disease progression. Although a number of K^+^‐sensitive imaging probes have been reported in this endeavour, to date these have been almost exclusively fluorescent imaging based (with associated poor signal penetration) [43, 44, 45], with, to the best of our knowledge, only a single report of a K^+^‐sensitive MRI probe (and this possessing only a very modest ∼5% relaxometric switch [46]. The generalised scheme of “water rigidification” was, accordingly, then translated to particles incorporating side wall hydrogen bonding benzo‐18‐crown‐6 (B18C6) moieties. These were observed to endow dramatic, and highly selective, cation‐binding‐induced relaxation switches, where marked changes in *T*_1_ are triggered at physiologically relevant potassium concentrations. This mechanistically new contrast switch constitutes a significant step towards the design and optimisation of ion‐responsive agents capable of supporting pathology‐relevant cation imaging.

## 2 Results and Discussion

### 2.1 Enhancing MRI Contrast by Promoting OS Effects

Native MSNs were synthesised by a modification of the Stöber process in a double delay co‐condensation procedure as previously reported by us [13]. During the co‐condensation reaction, the MSN amine functionalisation with (3‐aminopropyl)triethoxysilane (APTES) was achieved through addition at both 10 min and 1 h time intervals, (Schemes S1–S3) enabling a homogenous integration of anchor points along the MSN pore channel. These APTES doping levels can be synthetically tuned to generate either amine‐rich (NH_2_‐MSNs, Scheme S2) or silanol‐dominant (OH‐MSNs, Scheme S1) nanoparticles. In the absence of a high concentration of APTES, the pore channel is largely retained as native silanols (Scheme S1), with prior reports estimating the hydroxyl density within the MCM‐41 architecture to be ∼4.0 nm^−2^ (average silanol spacing ∼ 0.5 nm) [37, 47]. The introduction of (3‐mercaptopropyl)trimethoxysilane (MPTES) during the synthesis in place of APTES enabled the generation of thiolated particles (SH‐MSNs, Scheme S3). For all these formulations, 0.3% APTES dopant levels were retained across all particle formulations (NH_2_‐, OH‐ or SH‐MSNs) for subsequent chemical modification with either 2,2’,2″,2″‐(1,4,7,10‐tetraazacyclododecane‐1,4,7,10‐tetrayl)tetraacetic acid (DOTA), for *q* = 1 contrast generation (post metalation with Gd^3+^), or a pre‐metalated (Gd^3+^/Eu^3+^) ((1,4,7,10‐tetraazacyclododecane‐1,4,7‐triacetic acid)picolinoyl)glycinate, Gd/EuDO3AGlyPic, for *q* = 0 analyses (Schemes S10,S11). The aminated, thiolated and native particles exhibited high levels of colloidal stability (polydispersity index, PDI, <0.1) and average hydrodynamic sizes of 91.6 ± 8.0 nm (NH_2_‐MSNs), 91.2 ± 2.0 nm (SH‐MSNs) and 100.1 ± 3.0 nm (OH‐MSNs) respectively as recorded for 1 mg mL^−1^ dispersions in water (Figures S1,S2). Nitrogen adsorption‐desorption measurements, employing a Barrett‐Joyner‐Halenda (BJH) model for analysis, confirmed the mesoporous nature for all formulations, with associated pore diameters ranging from 2.6–3.5 nm (Figure S3). All modifications were tracked by DLS, possessing changes in ζ‐potential that align with functional group p*K*_a_ as recorded for 1 mg mL^−1^ dispersions of the particles across a full pH range (pH 3.0–10.0).

The native parent architectures (NH_2_‐/SH‐/OH‐) were functionalised with DOTA, or Gd/EuDO3AGlyPic, through a *N*‐hydroxysuccinimide‐mediated and base catalysed amide condensation with MSN‐bound amines (Scheme S4). Given the synthesis of amine anchor‐point modified MSNs known to be within the pore channel, we fully expect mesopore channel confined Gd‐chelate locality (consistent with prior reports) [13]. From a knowledge of particle size and TEOS/APTES ratio applied at the point of synthesis, we can estimate approximately 2000 amine anchor points available for macrocycle tethering per MSN (0.00065 amine / nm^2^; see SI), with this kept consistent across all particle formulations. For the Gd‐DOTA modified MSNs (i.e., non‐*q* = 0) post Gd^3+^ metalation of integrated DOTA moieties afforded the MRI active MSNs. We estimate an average of approximately 1800 Gd‐centres per MSN (Figure S4), correlating to a grafting density of 0.0006 chelated Gd^3+^/nm^2^, consistent with the prior determined amine grafting density. The *q* = 1 MSNs were denoted as NH_2_‐Gd‐MSNs, OH‐Gd‐MSNs, and SH‐Gd‐MSNs, with Gd‐chelation conducted prior to any further chemical modification to ensure the absence of any non‐specific Gd‐interaction with the introduced acidic moieties (Scheme S4). Quantitative Gd^3+^ chelation was confirmed by inductively coupled plasma mass spectroscopy (ICP‐MS) analyses (Figure S4) with control samples (chelate free) exhibiting a negligible presence of non‐specific Gd^3+^ (<3% “free” Gd^3+^ resolved by ICP‐MS, SI). These observations of tightly controlled Gd^3+^ were independently verified by both EDTA and arsenazo(III) titrations (Figures S5–S7). For *q* = 0 analyses, GdDO3AGlyPic was integrated into amine‐dominant MSNs by the aforementioned carbodiimide coupling approach and denoted NH_2_‐Gd(*q* = 0)‐MSNs.

The residual free amines in the amine‐dominant MSNs were subsequently converted to carboxylic acid moieties (COOH‐Gd‐MSNs/COOH‐Gd(*q* = 0)‐MSNs) in a one‐step reaction with an excess of succinic anhydride (Scheme S5). A subsequent carbodiimide coupling reaction with histamine was utilised to generate imidazole modified MSNs (Im‐Gd‐MSNs, Scheme S6). A peroxide oxidation of thiol‐dominant MSNs was employed to produce sulfonate‐dominant (SO_3_H‐Gd‐MSNs, Scheme S7). Im‐Gd‐MSNs and SO_3_H‐Gd‐MSNs were generated for only Gd‐DOTA (*q* = 1) modified MSNs. Transmission electron microscopy (TEM) resolved images (Figure S8) are fully consistent with expectations, as are dynamic light scattering (DLS) and ζ‐potential characteristics; functional group p*K*_a_ can be entirely mapped through analysis of ζ‐potential trends across pH 3.0–10.0 for all modified particles (Figure S9). Attenuated total reflectance infra‐red (ATR‐IR, Figure S10) spectroscopic analyses for each of the MSN formulations confirmed both the removal of the surfactant template (absence of peaks at 2850 cm^−1^ and 2920 cm^−1^) and the maintained integrity of the underlying silica architecture (strong stretch at 1060 cm^−1^).

**FIGURE 2:**
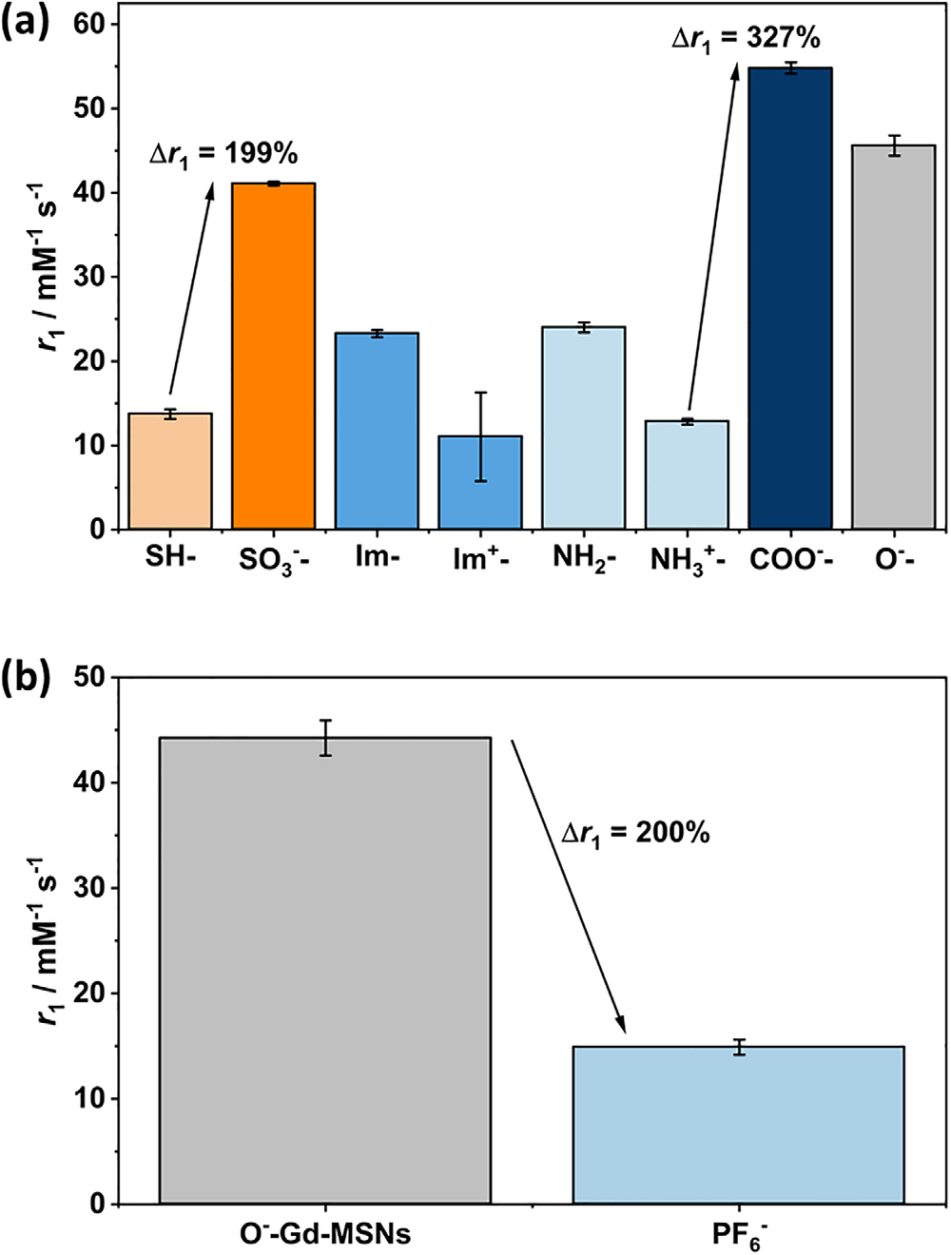
(a) The effect of introducing strong kosmotropic side wall modifications on Gd‐MSN *r*_1_. Notably, this can be achieved in a one‐step reaction from the parent architecture; a peroxide oxidation to generate SO_3_^−^‐Gd‐MSNs from SH‐Gd‐MSNs, or succinic anhydride coupling with excess amines to generate COO^−^‐Gd‐MSNs from NH_3_^+^‐Gd‐MSNs (with resolved percentage switches annotated). (b) *r*_1_ values for hydroxylated MSNs before and after adding the chaotropic PF_6_^−^ ion (10 mM). The significant ∼200% reduction in *r*_1_ highlights the effectiveness of PF_6_^−^ in disrupting interfacial water order. Error bars correspond to ±1 s. d. across three independent repeat measurements. *r*_1_ values were recorded by dispersing the MSNs (1 mg mL^−1^) in water at pH 7.0 (at 1.4 T and 298 K).

To investigate how these internal interface modifications affected MSN supported image contrast (through changes in internalised water viscosity and OS relaxation; see below), longitudinal relaxivities (for *q* = 1 MSNs) were analysed for each formulation. These were acquired by dispersing the particles in water at pH 7.0 (unless otherwise indicated) and recorded by nuclear magnetic resonance (NMR, at 1.4 T). Observations across a range of side wall modifications are shown in Figure 2a. It is initially notable that native silanol (O^−^–Gd‐MSNs) particles yield an associated high *r*_1_ = 45.6 ± 1.2 mM^−1^ s^−1^, likely indicating significant ordering of water molecules at the hydroxylated surface. To confirm this, the chaotropic PF_6_^−^ ion (at 10 mM), known to disrupt water structure [48], was added and observed to induce a 200% decrease in *r*_1_ (Figure 2b), an observation fully consistent with a reduction in interfacial water ordering. Similar structure directing effects were also observed for analogous anionic water ordering side‐wall functional groups (e.g., COO^−^–/SO_3_^−^‐, Figure 2a). Notably, carboxylated side wall MSNs exhibit a >300% enhanced relaxivity over their parent amine analogue for equivalent functional group loading density. Carboxylates are known to instil significant amounts of local water ordering [36, 49, 50, 51], and, consistent with this, COO^−^‐Gd‐MSNs possess *r*_1_ values that are exceptionally high (54.8 ± 0.7 mM^−1^ s^−1^) at this field strength. A comparable effect is observed for the SO_3_^−^ modified MSNs, with a 199% enhancement in *r*_1_ relative to the parent SH‐Gd‐MSNs (Figure 2a). Charge neutral side wall motifs (including Im‐ and NH_2_‐Gd‐MSNs, see Figure 2a, achieved through a control of solution pH i.e., recording *r*_1_ above the p*K*_a_ of Im/NH_2_) exhibit markedly lower relaxivities than their anionic counterparts, an observation consistent with reduced water ordering as effected by the amine electron lone pair over the electrostatic ion [52]. Interestingly, MSN side walls with NH_3_^+^‐/Im^+^‐ groups (recording *r*_1_ below the *p*K_a_ of Im/NH_2_) were shown to dramatically suppress longitudinal relaxivities from the hydroxyl side‐wall baseline (Figure 2a). This observation is very likely a reflection of the developed electrostatic association between the anionic chelate/side wall, dramatically reducing water exchange [53].

To further demonstrate that these observations arise from MSN side wall water ordering effects, control *r*_1_ measurements for Gd‐DOTA in water were compared to those for Gd‐DOTA in solutions of K_2_SO_4_ (5 mM, kosmotropic ion) or NaPF_6_ (10 mM, chaotropic ion, Figure S11). Predictably, there were no changes in *r*_1_ given that local water viscosity cannot be tuned for the molecular analogue. It should be noted that all generalised sidewall effects are independent of mesopore diameter and map equivalently onto analogous dendritic large pore mesoporous silica nanoparticles (DMSNs, Figures S20–S28) [54].

In building a picture of large and modulatable OS effects for these particulate configurations, we then silenced IS contributions to relaxivity entirely by running the same analyses with integrated *q* = 0 complexes (in which IS contributions are innately zero, see Figure S12 where *r*_1_ (GdDO3AGlyPic) = 1.6 ± 0.4 mM^−1^ s^−1^) [55]. Specifically, we integrated the *q* = 0 GdDO3AGlyPic ligand into the MSN scaffold, first confirming that *q* = 0 was retained by the Eu‐doped complex within the particle scaffold under side wall modification (Figures S16,S17). Interestingly, relaxivities were exceptionally high for kosmotropic functionalised side walls, and chemically tuneable as for the *q* = 1 analogues and down‐tuned through the addition of a chaotrope to solution (Figure S12). Specifically, for COO^−^‐Gd(*q* = 0)‐MSNs the recorded *r*_1_ was 49.9 ± 2 mM^−1^ s^−1^ at 1.4 T, i.e., 91% of the value recorded for the analogous Gd‐DOTA modified carboxylate MSNs (Figure S12). This represents a *q* = 0 contrast generating capability that is entirely unprecedented.

**FIGURE 3:**
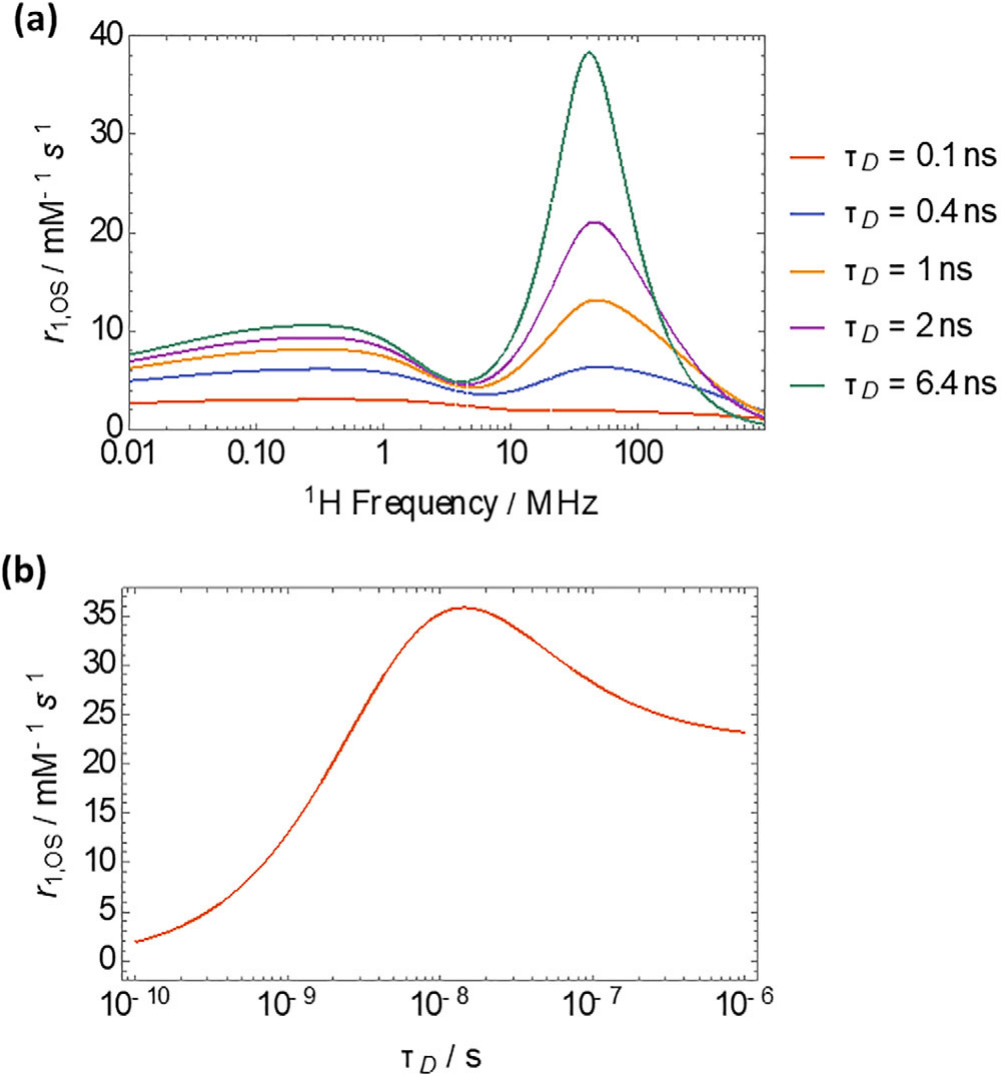
An analytical treatment as given by SBM theory showing how differences in the diffusion correlation time affects the observed OS contribution to *r*_1_. (a) The effect of changing τ_D_ on the OS contribution to *r*_1_ across a range of different ^1^H frequencies. The distance between the paramagnetic Gd‐centre and the water proton was set at a = 0.5 nm (i.e., based on predicted distances between adjacent silanol groups, and therefore an estimate for the distance between the paramagnetic chelate and kosmotrope motif). (b) The effect of τ_D_ on the OS contribution to *r*_1_ at 60 MHz (a = 0.5 nm). As τ_D_ is increased from bulk (i.e., water diffusion slowed) then significant OS mediated contributions to *r*_1_ can be achieved.

It is unequivocal, then, that (tuneable) OS effects dominate the resolved contrast‐generating characteristics of these particle formulations. We can further apply a theoretical SBM treatment (SI) to show that sensible modulations in the water diffusion coefficient (and thus *τ*_D_) can fully account for the observed enhancements in *r*_1_ (Figure 3a,b); increasing *D* (from 2.3 x 10^−9^ m^2^ s^−1^, i.e., for bulk water at 298 K, to 2.3 x 10^−11^ m^2^ s^−1^, quantifications also entirely aligned with independent work) [38, 56, 57], causes *r*_1_^OS^ to dramatically increase (ranging from a 70–2000% increase, Figure 3a,b) for a side wall hydroxylated MSN, in line with expectations. We can also show that modulations in relaxivity cannot be sensibly accounted for by variance in chelate‐side wall mechanical coupling and thus local rotation (see discussion in the SI) [58]. Similarly, a 10‐fold change in *τ*_M_ i.e., from *τ*_M_ = 208 ns, a realistic estimation for Gd‐DOTA [58], to *τ*_M_ = 19.4 ns, a value chosen specifically to optimise *r*_1_, again, resolved changes in *r*_1_^IS^ insignificant in magnitude to those highlighted in this work. Additionally, given that typical water exchange rates lie well above optimal for Gd‐MSN adducts [59], the actual change in *r*_1_ is likely to be even smaller that theoretically derived. It should be noted, however, that longitudinal relaxivity is, of course, a composite sum of IS, SS and OS components; IS and SS contributions constitute a constant background (IS quantified by *q* = 0 analyses herein). We consider that the average silanol (and modifications therein) spacings fall beyond the local paramagnetic chelate sphere (for SS) [47], and assign changes in *r*_1_ across different side wall modifications to realistic but significant modulations in *τ*_D_ [35, 56, 57].

**FIGURE 4:**
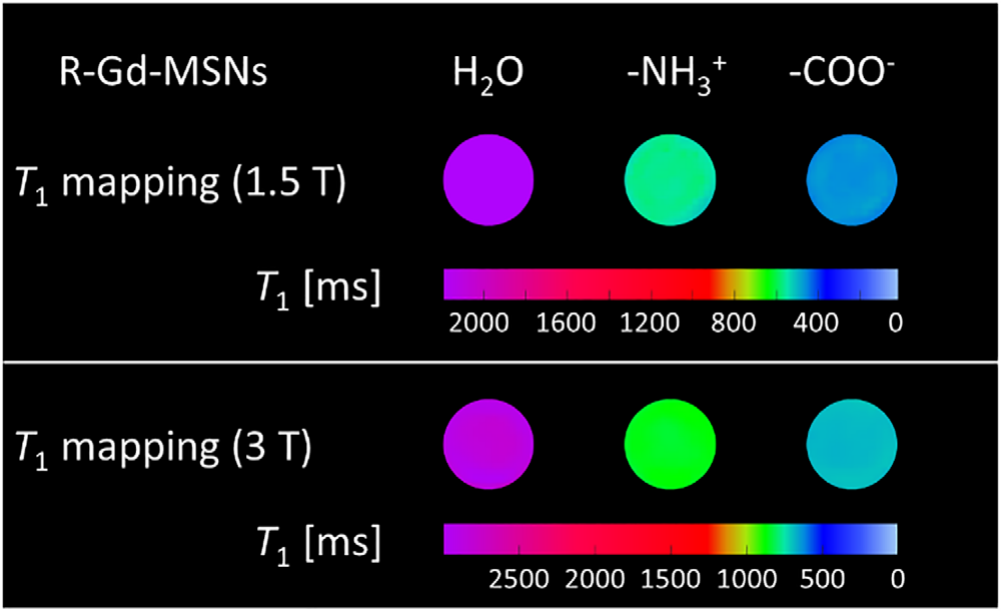
*T*_1_‐weighted MRI maps collected using clinical scanners for the NH_3_^+^‐Gd‐MSNs and COO^−^‐Gd‐MSNs recorded at both 1.5 T and 3 T. The corresponding *T*_1_ times at 1.5 T are 2774 ± 29 ms for water, 565 ± 7 ms for the NH_3_^+^‐Gd‐MSNs and 466 ± 7 ms for the COO^−^‐Gd‐MSNs. At 3 T the corresponding *T*_1_ times are 2873 ± 42 ms for water, 857 ± 12 ms for the NH_3_^+^‐Gd‐MSNs and 686 ± 6 ms for the COO^−^‐Gd‐MSNs. All measurements were for a consistent Gd‐concentration of 0.04 mM loaded within the MSNs (ICP‐MS confirmed) in water at pH 7.0.

These relaxometric trends were mapped by translationally‐relevant clinical MRI scanners, at both 1.5 T and 3 T (Figure 4, Figure S18), using a clinical body imaging *T*_1_‐mapping sequence typically applied for whole body imaging. Large differences in image contrast for the NH_3_^+^‐ and COO^−^‐Gd‐MSNs were clearly resolvable and consistent with the relaxivities as resolved by NMR (Figure 4 and Figure S18), with general trends correlating tightly with the kosmotropic effects on *r*_1_ previously noted. It should be highlighted that the Gd‐concentrations remained constant across all particle configurations (ICP‐MS resolved, [Gd] = 0.04 mM).

### 2.2 K^+^‐responsive MRI Contrast

**FIGURE 5:**
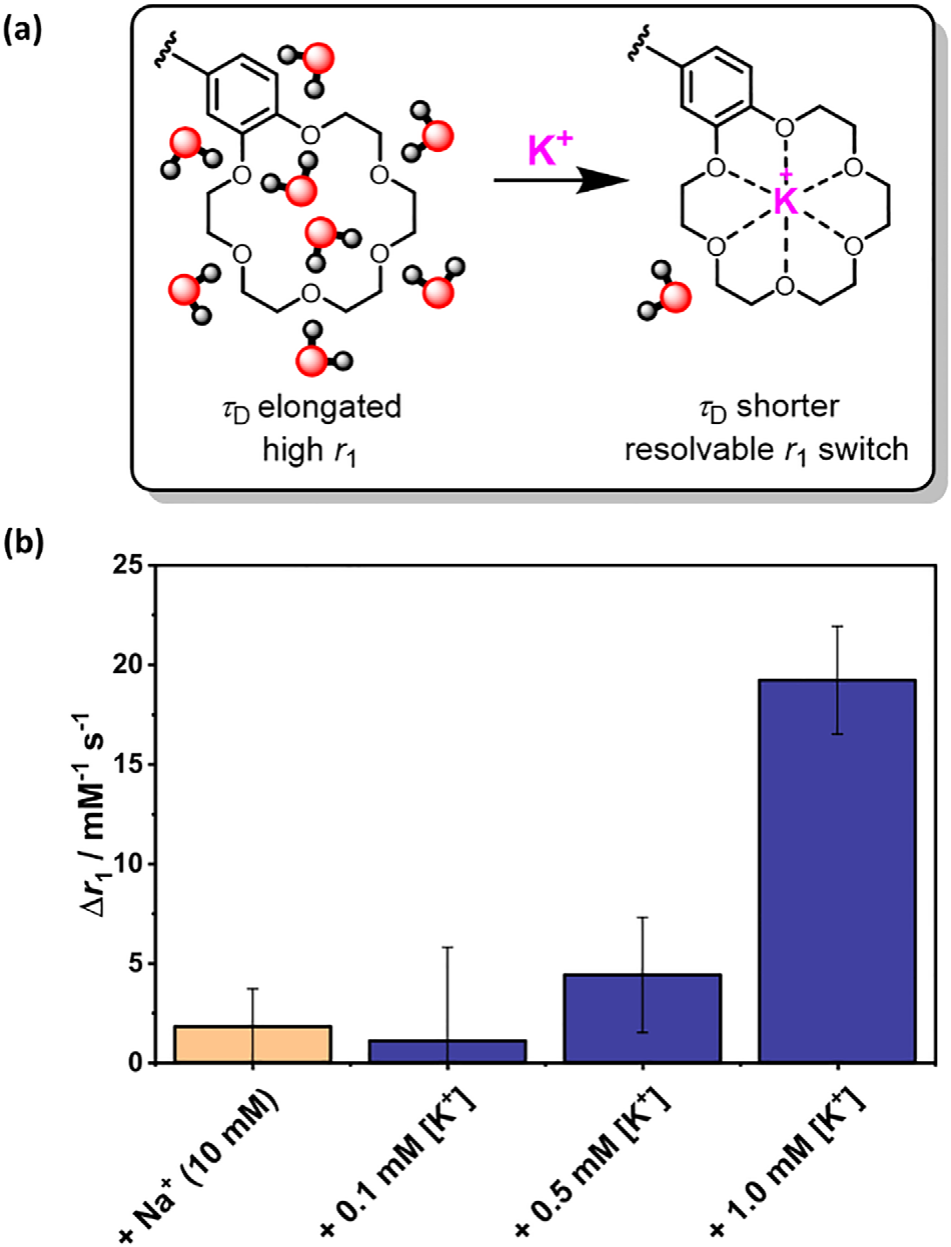
(a) A schematic summarizing the differences in CE‐mediated water ordering on complexation with K^+^. (b) Change in *r*_1_ (calculated from the initial *r*_1_ for the CE‐MSNs—final *r*_1_ on incubation with K^+^) for the CE‐MSNs (recorded at 1.4 T and 298 K) on addition of NaCl (10 mM), or when in the presence of KCl (varying from 0.1–1.0 mM). The switching magnitude of *r*_1_ scales with progressive increases in K^+^ concentration and is at a maximum when [K^+^] = 1 mM. All switches in *r*_1_ are comfortably image resolved, see Figure 6. Error bars correspond to ± 1 s d. across three independent repeat measurements.

Thus far it is evident that a highly localised control over water mobility can underpin significant enhancements and modulations in MR image contrast. We expected the basicity of a polyether scaffold to support high levels of hydration in the absence of a (cationic) host [60, 61], thereby restricting water mobility within a confined space, decreasing *D*, and promoting an elongation of *τ*_D_ that should be selectively switched off in the presence of a specific cation recruitment as the host basicity is reallocated (Figure 5a) [60, 61]. Motivated by this, we generated benzo‐18‐crown‐6 (B18C6) modified MSNs (Scheme S8, S9 and Figures S29–S31), denoted crown ether‐MSNs, CE‐MSNs). This crown ether has a well‐known strong association with water and a high selectivity for the complexation of K^+^ [60, 61]. Incubation of the CE‐MSNs in low mM K^+^‐concentrations (0.1–1.0 mM) leads to a progressive and significant downward switch in *r*_1_ (∆*r*_1_ = 19.2 mM^−1^ s^−1^, i.e., 40%, at 1.0 mM K^+^, Figure 5b) even at low K^+^, observations we directly assign to host‐guest complexation and the accompanying substantial decrease in water organisation (Figure 5a). The selectivity of B18C6 for K^+^ over Na^+^ is particularly striking, with no changes in relaxivity observed in the presence of NaCl (10 mM, Figure 5b), see Figure S32 for more detailed comparisons with other alkali metal cations). Pleasingly, these effects are entirely retained with *q* = 0 paramagnetic particles with significant (25%) switches in *r*_1_ achieved only by the host‐guest recognition with K^+^, consistent with the *q* = 1 analyses. Achieving analogous relaxivity switching for *q* = 0 particles, where IS effects have been silenced, underpins the potency that manipulating the OS relaxation pathway can have on CA performance.

**FIGURE 6:**
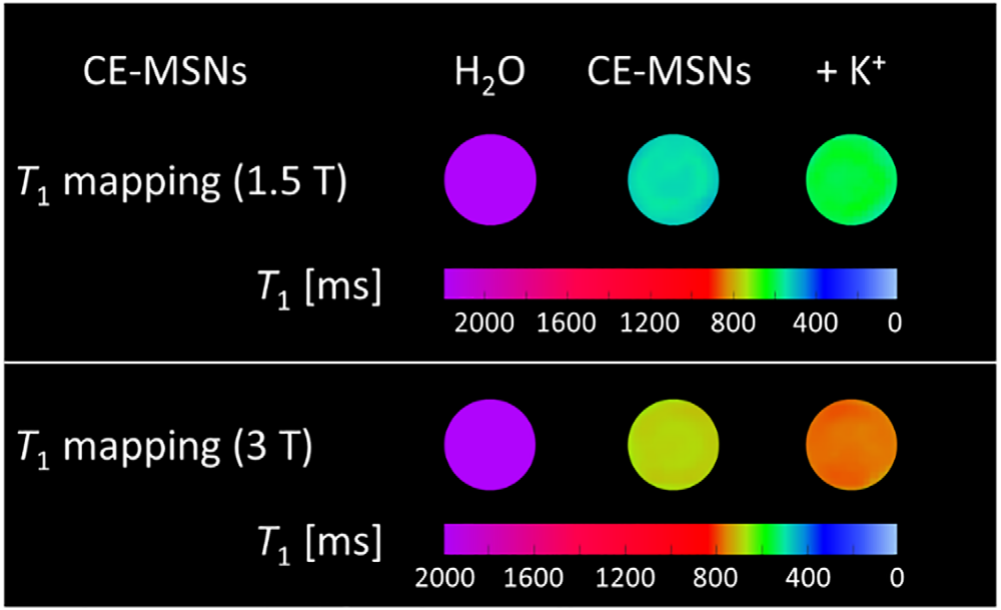
MRI *T*_1_ maps collected using clinical scanners for the crown ether modified MSNs in the absence and presence of potassium (KCl, 10 mM), as recorded at 1.5 and 3 T (at 298 K). For the CE‐MSNs, in both the presence and absence of K^+^, the Gd^3+^ concentration was maintained at 0.030 mM. The corresponding *T*_1_ times at 1.5 T are 2774 ± 29 ms for water, 543 ± 7 ms for the CE‐MSNs in water and 609 ± 9 ms for the CE‐MSNs in the presence of K^+^. The corresponding *T*_1_ times at 3 T are 2873 ± 42 ms for water, 691 ± 5 ms for the CE‐MSNs in water and 772 ± 6 ms for the CE‐MSNs in the presence of K^+^.

The above relaxivity trends are, of course, mapped a across more translationally‐relevant imaging, as acquired at both 1.5 T and 3 T (Figure 6, Figure S33, S34), using the same clinical body imaging *T*_1_‐mapping sequence (*vide supra*). The image contrast generated by the CE‐MSNs (at a constant [Gd]) is clear at both field strengths (Figure 6), with an associated switch in image contrast in the presence of 10 mM K^+^, mirroring the prior discussed relaxivity assessments. It is notable that these crown‐doped MSNs exhibit cation triggered *r*_1_ switches that are large in comparison to nearly all prior cation‐responsive paramagnetic agents (see Figure S34 for Δ*r*_1_ values at 1.5 and 3 T), and, notably, very substantially larger than prior reported for any K^+^‐responsive MR probe [46, 62]. It is, additionally, worth noting that these cation concentrations are entirely relevant to K^+^‐related pathology; serum K^+^ concentrations typically range from 3.5–5.5 mM [63, 64, 65]. The value of spatially resolved K^+^ contrast imaging (intra‐ and extracellular) has been prior noted [66, 67] and we believe the toolbox presented herein represents a valuable (and mechanistically entirely new) addition to this capability [68].

## 3 Conclusion

Gd‐centres are known to engender water relaxation by three distinct mechanisms; IS relaxation mediated through the direct dipolar coupling of electron and nuclear spin, SS arising from chelate hydration, and OS relaxation that is dependent on water diffusing nearby the Gd‐chelate [26]. The latter has typically been neglected given that water diffusing near a molecular paramagnetic‐centre has a diffusion time that correlates with bulk (far from the Larmor resonance, and thus of limited contribution to *r*_1_). Nano‐confined water has been prior reported to be significantly different to bulk [21], being notably more viscous at some interfaces [33, 34, 35, 36, 37]. It has, specifically, been shown that the diffusion coefficient can be stretched by more than two orders of magnitude at highly hydrated silica interfaces [38]. We show unequivocally in this work that the OS relaxation pathway can be heavily promoted when water is dramatically slowed through its interaction with kosmotropically modified side walls. Whilst the contribution of IS/SS effects cannot be entirely excluded, they constitute a constant background against a modulated OS contribution. The latter are not only large enough to support very high levels of relaxivity/contrast for *q* = 1 paramagnetic systems, but can also support unprecedented levels of contrast for *q* = 0 configurations. These effects can pleasingly be mapped out across independent NMR and imaging configurations, reversed through the addition of water‐disrupting chaotropes to solution and supported by sensible modulations in diffusion parameters within a theoretical framework. For crown‐ether modified scaffolds, very substantial image contrast responses to physiologically‐relevant cation concentrations are, then, afforded. We envisage that the basic chemical principles outlined here could be leveraged in the production of highly effective and/or responsive MRI contrast agents generally. In considering clinical translatability, accessible silanol chemistry provides a means by which nanoparticulate silica interfaces are readily modified to extent blood circulation times or to introduce targeting vectors (controlling accumulation site), or both [15]. Further applications could also consider both the integration of a ratiometric response enabling a direct quantitative assessment of tissue ion imbalance, and/or peripheral targeting agents. The former would enable a quantitative understanding of ion‐associated pathology and could be achieved through integration of a background reporter probe within the particle (*e.g*., an unresponsive *T*_2_‐active SPION core, alternative metal chelate or integrated spin‐active nucleus), measuring relaxation rate ratios on cation exposure, or analyses at different field strengths [7, 69].

## Author Contributions

**Connor M. Ellis**: investigation, writing – original draft. **James P. Smith**: investigation, writing – original draft. **Matthew F. Allen**: investigation. **Ferenc E. Mózes**: investigation, formal analysis. **Stephen Faulkner**: writing – original draft. **Jason J. Davis**: conceptualization.

## Conflicts of Interest

The authors declare no conflicts of interest.

## Supporting information




**Supporting File**: anie72596‐sup‐0001‐SuppMat.docx.

## Data Availability

The data that support the findings of this study are available from the corresponding author upon reasonable request.
