# Supplementary material for: Slowing Down Water: Enhanced and Cation‐responsive MRI Contrast
Source: Angew Chem Int Ed Engl. 2026 May 20;65(29):e5723493. doi: 10.1002/anie.5723493 (PMC13360556; doi:10.1002/anie.5723493)
Supplement: Supplementary file 1 — Supporting File: anie72596‐sup‐0001‐SuppMat.docx. [file ANIE-65-e5723493-s001.docx]

Supplementary Information

**Slowing down water: enhanced and cation-responsive MRI contrast**

Connor M. Ellis,^a^ James P. Smith,^a^ Matthew F. Allen,^a^ Ferenc E. Mózes,^b^ Stephen Faulkner,^a^ and Jason J. Davis*^a^

1. Department of Chemistry, University of Oxford, South Parks Road, Oxford, OX1 3QZ, UK
2. Oxford Centre for Clinical Magnetic Resonance Research, Radcliffe Department of Medicine, University of Oxford, Level 0, John Radcliffe Hospital, Oxford, OX3 9DU, UK

*E-mail:* [*Jason.davis@chem.ox.ac.uk*](mailto:Jason.davis@chem.ox.ac.uk)*; Fax: +44 (0)1865 272 690; Tel: +44 (0)1865 275 914*

**Experimental Section**

**Chemicals:**

2,2',2''-(10-(2-((2,5-dioxopyrrolidin-1-yl)oxy)-2-oxoethyl)-1,4,7,10-tetraazacyclododecane-1,4,7-triyl)triacetic acid (DOTA-NHS-ester) was purchased from CheMatech. Ultrapure water (Millipore) with a resistivity of 18.2 MΩ∙cm was used throughout. Experiments were performed using commercially purchased reagents and solvents without further purification unless otherwise stated.

**Characterisation:**

A Malvern Zetasizer Nano with a 532 nm laser as the light source was used to perform the dynamic light scattering analysis (DLS) to acquire both hydrodynamic size and zeta potential measurements. The samples were prepared for DLS by dispersing the sample (ca. 1 mg mL^-1^) in the desired solvent for analysis. The corresponding Gd^3+^ concentrations were calculated (for every MSN formulation) and verified using inductively coupled plasma mass spectrometry (ICP-MS) analysis (Perkin Elmer NexION 2000B). The samples were prepared by hydrolysing the nanoparticles in 3 mL HNO_3_ (70%) overnight. The samples were then adjusted to a final dilution of 0.3 M HNO_3_ using 18.2 MΩ deionised water. The calibration curve was obtained using an external calibration analysis (a series of standards of known concentrations forming a linear plot for calibration, diluted from a 10 ppm stock standard), prepared using SPS-SW2 standards (Spectrapure Standards, Oslo). During the ICP-MS measurements, samples without Gd^3+^ loading were set as the baseline (*i.e.*, as the concentration of 0 mM), with actual Gd^3+^ quantifications determined by subtracting the reference points. Control reactions with MSNs in the absence of DOTA resolved highly specific (> 97%) chelation as resolved by ICP-MS analyses. Specifically, control reactions confirmed 0 mM and 0.0009 mM of Gd^3+^ associated with the particles in the absence of DOTA for the parent NH_2_-MSN and native OH-MSN architectures respectively. These are 0% and 2.6% of the corresponding ICP-MS concentrations for the NH_2_-Gd-MSNs (0.045 mM) and OH-Gd-MSNs (0.034 mM) respectively (confirming the effective removal of any non-chelated Gd^3+^). Transmission electron microscopy (TEM) images were acquired using a FEI Tecnai-T12 (USA) operated at 120 kV. The samples for TEM were prepared by depositing a drop of the aqueous colloidal suspension (*c.a.* 0.1 mg mL^-1^) onto a copper grid. Attenuated total reflectance infrared (ATR-IR) analysis was conducted on an IRTracer-100 (Shimadzu) spectrometer. Barrett–Joyner–Halenda (BJH) nitrogen adsorption-desorption analyses were acquired using a Micromeritics TriStar II PLUS surface characterisation analyser.

Flash column chromatography was undertaken using Merck silica gel 60, thin layer chromatography using Merck silica coated aluminium plates (60G F254) and visualised using UV-light (254 nm) and permanganate or ninhydrin stains. Purification of discrete lanthanide complexes by dialysis was undertaken using Float-A-Lyzer ® G2 dialysis tubes (500 MWCO) equipped with regenerated cellulose. Milli-Q, ultrapure, water was used and compounds dialysed in 5 L beakers. Dialysis was completed over a duration of two days with a total of eight water changes.

NMR spectra were collected on a Bruker Avance III HD nanobay NMR equipped with a 9.4T magnet (1H 400.2 MHz, 13C 100.6 MHz). All measurements were conducted at 298 K unless stated otherwise. Spectra were processed using Bruker Topspin 4.1.1 and MestReNova x64 software packages. Signals for chemical shift were reported with reference to the residual solvent peak (CDCl3, δ = 7.26 ppm; DMSO-d6, δ = 2.50 ppm; CD3CN, δ = 1.94 ppm) and reported in the following format: chemical shift, multiplicity (s – singlet, d – doublet, t – triplet, q – quartet, sx – sextet, sp – septet, o – octet, m – multiplet), coupling constant (J, Hz), integration, and assignment of proton relative to spectrum.

High resolution mass spectrometry (BioAccord 2): flow injection analysis was performed on an ACQUITY I-Class PLUS UPLC System (Waters, Milford, MA, USA) coupled to an ACQUITY RDa mass spectrometer (Waters, Milford, MA, USA) equipped with an ESI probe, in positive ion mode. The flow rate was set to 0.300 mL/min using a 50% methanol(aq) + 0.1% formic acid eluent. Scan parameters were set as follows: analyser mode, full scan; scan range, 50-2000 m/z; scan rate, 2 Hz; cone voltage, 30 V; capillary voltage, 1.5 kV; desolvation temperature, 550°C; and intelligent data capture, on.

Photophysical measurements were undertaken in quartz cuvettes with a path length of 1 cm at room temperature. Discrete complexes were prepared at a concentration of 50 μM in water and deuterium oxide. Eu doped MSN particles were prepared at a concentration of 20 mg mL^-1^ in both buffered water and deuterium oxide solutions at pH/pD 4.0 (Acetate buffer, 30 mM) and pH/pD 7.0 (HEPES buffer, 30 mM). Absorption spectra in the UV-Visible region were collected on a thermoscientific Genesys 50 UV-Visible spectrophotometer. Steady-state and time-gated emission and excitation spectra were collected on a Horiba Jobin Yvon Fluorolog® 3-12 Fluorometer utilising the FluorEscence software package (v3.5), and equipped with a double grating emission monochromator and a Hamamatsu R928 detector. Corrected emission (S1c channel) was reported throughout as the emission output. For time-gated emission spectra where the excitation was at 280 nm, a 495 nm long pass filter was used (FGL495S, ThorLabs) the excitation and emission slit widths were 10 nm and 1 nm with a flash count of 2 and time gate of 50 μs, while excitation at 393 nm utilised 20 nm, 5 nm, flash count of 5 and a 50 μs gate, both were corrected by subtracting a solvent blank under the same conditions. Lifetime measurements employed excitation and emission slits of 8:2 nm, and 15:7.5 nm at excitation wavelengths of 280 nm and 393 nm respectively, monitoring Eu^3+^ luminescence via the ^5^D_0_ → ^7^F_2_ (615 nm) transition. Lifetime measurements were carried out over a three-run average to minimise noise and were subsequently fit to exponential decay functions. Photophysical data were processed using OriginPro (Version 2024b. OriginLab Corporation, Northampton, MA, USA.). The number of bound water molecules (*q*) was determined using the Horrocks equation (modified), $q=A(k_{H_{2}O}-k_{D_{2}O}-B)$, where A = 1.2 ms, B = 0.25 ms^-1^, and $k_{H_{2}O}$/$k_{D_{2}O}$ are the observed luminescence rates (ms^-1^) in H_2_O and D_2_O respectively.^1^

The longitudinal relaxation times were recorded at 1.4 T using a Spinsolve Benchtop NMR (Magitrek) at room temperature (298 K unless otherwise stated). The samples were dispersed in ultrapure water at the desired Gd^3+^ concentration (dilutions of 1 mg mL^-1^, 0.5 mg mL^-1^, 0.25 mg mL^-1^ and 0 mg mL^-1^) with the longitudinal relaxation rate (1/$T_{1}$) plotted against the concentration of Gd^3+^ (mM, as determined by ICP-MS analyses for all MSN formulations). The relaxivity (mM^-1^ s^-1^) was then obtained from the slope of the subsequent linear fit, with the goodness of fit parameter ($R^{2}$) shown to be $\geq$ 0.91 in all cases. The error assessments accounted for both their (i) deviations from the linear regressions and (ii) standard deviations from three replicate measurements, with the larger of the two taken as the representative $r_{1}$ deviation.

MR imaging was performed on scanners routinely used for both clinical research and to provide a cardiology service to NHS patients: specifically, Siemens 3 T Prisma and Siemens Avanto Fit 1.5 T systems, both sited in a temperature-controlled, remotely monitored room. For sample preparation, MSN Gd-dopant levels were initially resolved by ICP-MS, to ensure that the dispersed particles (in water) had a consistent [Gd] = 0.04 mM across each sample, supporting quantitative comparisons. The samples were placed on the patient table with two large quality control fluid phantoms placed nearby for coil loading purposes: all imaging was undertaken using the body coil for RF transmit, and the spine array and 18-channel coils for RF receive on each scanner. After the acquisition of localisers and an automated shimming routine, shortened modified Look-Locker imaging (ShMOLLI) $T_{1}$ maps were acquired as described previously,^2^ with a 192 x 144 acquisition matrix, 384 x 288 reconstruction matrix, 360 x 270 mm^2^ FOV, 8 mm slice thickness, TE = 1.01 ms, TR = 2.05 ms, 35° readout flip angle, GRAPPA acceleration factor of 2 with 24 reference lines, 6/8 partial Fourier, inversion times 100 ms, 1100 ms, 2100 ms, 3100 ms, 4100 ms, 180 ms, 260 ms. The parameters of this *T*_1_ mapping sequence match those used routinely for human cardiac imaging.

**Preparation of double-delay 0.3 % aminated MSNs by a modified Stöber method [1]:**

**Scheme S1.** Synthesis of 0.3 % aminated MSNs (OH-MSNs) by a double-delay co-condensation approach.

1.77 mmol of cetyl trimethylammonium bromide (CTAB) and 6.9 mmol of triethanol amine (TEA) were dissolved in a water/ethanol mixture (1.88:16.2 mL EtOH:H_2_O) with the solution vigorously stirred at 80 °C for 20 min. Then, 5.18 mmol of tetraethylorthosilicate (TEOS) was added dropwise (1 mL/min). 10 min after the addition of TEOS, 2.6 μL of aminopropyltriethoxysilane (APTES) was added to the solution. 60 min after the initial TEOS addition, 2.23 μL of TEOS and 2.6 μL of APTES were added with the reaction continued to be vigorously stirred at 80 °C for a total reaction time of 2 h. The reaction was subsequently cooled down to room temperature, the particles collected by centrifugation (13,500 rpm, 20 min) and then purified using centrifugation in EtOH at 13,500 rpm for 20 min (x2). The mixture was then sonicated for 30 min in acidic EtOH (10 vol%) to remove all the toxic surfactant CTAB template and washed a further two times with EtOH. Finally, the nanoparticles were collected by centrifugation and dried under vacuum overnight to afford the double delay 0.3 % aminated MSNs (OH-MSNs).

**Preparation of double-delay 10 % aminated MSNs by a modified Stöber method [2]:**

**Scheme S2.** Synthesis of 10 % aminated MSNs (NH_2_-MSNs) by a double-delay co-condensation approach.

1.77 mmol of cetyl trimethylammonium bromide (CTAB) and 6.9 mmol of triethanol amine (TEA) were dissolved in a water/ethanol mixture (1.88:16.2 mL EtOH:H_2_O) with the solution vigorously stirred at 80 °C for 20 min. Then, 5.18 mmol of tetraethylorthosilicate (TEOS) was added dropwise (1 mL/min). 10 min after the addition of TEOS, 76 μL of aminopropyltriethoxysilane (APTES) was added to the solution. 60 min after the initial TEOS addition, 145 μL of TEOS and 76 μL of APTES were added with the reaction continued to be vigorously stirred at 80 °C for a total reaction time of 2 h. The reaction was subsequently cooled down to room temperature, the particles collected by centrifugation (13,500 rpm, 20 min) and then purified using centrifugation in EtOH at 13,500 rpm for 20 min (x2). The mixture was then sonicated for 30 min in acidic EtOH (10 vol%) to remove all the toxic surfactant CTAB template and washed two more times with EtOH. Finally, the nanoparticles were collected by centrifugation and dried under vacuum overnight to afford the double delay aminated MSNs (NH_2_-MSNs).

**Preparation of double-delay 9.7 % thiolated/0.3 % aminated MSNs by a modified Stöber method [3]:**

**Scheme S3.** Synthesis of 9.7 % thiol/0.3 % aminated MSNs (SH-MSNs) by a double-delay co-condensation approach.

1.77 mmol of cetyl trimethylammonium bromide (CTAB) and 6.9 mmol of triethanol amine (TEA) were dissolved in a water/ethanol mixture (1.88:16.2 mL EtOH:H_2_O) with the solution vigorously stirred at 80 °C for 20 min. Then, 5.18 mmol of tetraethylorthosilicate (TEOS) was added dropwise (1 mL/min). 10 min after the addition of TEOS, 2.6 μL of aminopropyltriethoxysilane (APTES) and 73.4 μL (MPTES) were added to the solution. 60 min after the initial TEOS addition, 145 μL of TEOS, 2.6 μL of APTES and 73.4 μL MPTES were added with the reaction continued to be vigorously stirred at 80 °C for a total reaction time of 2 h. The reaction was subsequently cooled down to room temperature, the particles collected by centrifugation (13,500 rpm, 20 min) and then purified using centrifugation in EtOH at 13,500 rpm for 20 min (x2). The mixture was then sonicated for 30 min in acidic EtOH (10 vol%) to remove all the toxic surfactant CTAB template and washed two more times with EtOH. Finally, the nanoparticles were collected by centrifugation and dried under vacuum overnight to afford the double delay thiolated MSNs (SH-MSNs).

**General procedure for complexation with LnCl_3_: [4]:**

**Scheme S4.** Integration of MSNs with DOTA and subsequent chelation to afford the Gd-MSNs.

200 mg of the desired MSN parent analogue (*i.e.*, OH-, NH_2_- or SH-MSNs) were dispersed, under sonication, in 15 mL of N,N-dimethylformamide (DMF). Then, 5.25 μmol of DOTA-NHS ester and 150 μL of triethylamine were added to the reaction flask. The resultant mixture was stirred at r.t for 24 h. The nanoparticles were then collected by centrifugation at 13,500 rpm for 20 min and washed three times with EtOH to produce the desired DOTA modified MSNs (DOTA-MSNs). The obtained DOTA-MSNs were dispersed in 10 mL of EtOH to which 10 μmol of GdCl_3_.6H_2_O was added (or EuCl_3_.6H_2_O for *q* analyses). Then, vigorous stirring was carried out for 24 h at r.t. The Gd-doped nanoparticles (R-Gd-MSNs, where R corresponds to OH-, NH_2_- or SH-) were washed three times with EtOH and dried under vacuum.

**COOH modification of Gd-MSNs [5]:**

**Scheme S5.** Synthesis of carboxylate modified MSNs.

50 mg of NH_2_-Gd-MSNs were dispersed in 5 mL of DMF under sonication. 75 mg of succinic anhydride was added, and the solution stirred at room temperature for 24 h. The nanoparticles were washed three times with EtOH and then dried under vacuum to obtain the COOH-Gd-MSNs.

**Imidazole modification of Gd-MSNs [6]:**

**Scheme S6.** Synthesis of imidazole modified Gd-MSNs.

COOH-Gd-MSNs (22 mg) were resuspended in 2-(*N*-morpholino)ethanesulphonic acid buffer (MES, 15 mM, 2 mL, pH 6.0) to which EDC (15.06 mg) and NHS (22.33 mg) were added with the solution left to stir for 20 min at r.t. Histamine (5.39 mg) was added to the solution, with this left to stir overnight at r.t. The particles were collected by centrifugation (13,500 rpm, 20 min) and washed a further three times with EtOH before being left to dry overnight under vacuum.

**SO_3_H modification of Gd-MSNs [7]:**

**Scheme S7.** Synthesis of SO_3_H-modified Gd-MSNs.

Gd-loaded –SH modified MSNs (25 mg) were dispersed in 4 mL water to which 4 mL of H_2_O_2_ (30 %), was added and the obtained solution then stirred at room temperature overnight. After collecting the particles by centrifugation and washing with ethanol twice (13,500 rpm, 20 min), the MSNs were dispersed into 4 mL H_2_SO_4_ (0.2 M) for 2 h. The final product was washed twice with water using centrifugation (13,500 rpm, 20 min).

**Synthesis of NHS appended 18 crown 6 [8]:**

**Scheme S8.** Synthesis of benzo-18-crown-6 modified Gd-MSNs.

18 crown 6 carboxylic acid (150 mg, 0.421 mmol) was dissolved in anhydrous CH_2_Cl_2_ (ca. 15 mL), to which was added N-hydroxysuccinimde (NHS, 58 mg, 0.505 mmol), dicyclohexylcarbodiimide (95 mg, 0.463 mmol) and a catalytic amount of DMAP. The mixture was left to stir at room temperature for 3 hours, after which time the mixture was filtered and the filtrate diluted with CH_2_Cl_2_ (100 ml) and washed with H_2_O (2 x 15 ml) dried over MgSO_4_ and the filtrate concentrated in vacuo to afford the NHS appended 18 crown 6 as a white solid (143 mg, 0.316 mmol, 75 %).

^1^H NMR (400 MHz, CDCl_3_) δ 7.79 (dd, *J* = 8.5, 2.0 Hz, 1H), 7.59 – 7.52 (m, 1H), 6.95 – 6.85 (m, 1H), 4.25 – 4.09 (m, 4H), 4.01 – 3.85 (m, 4H), 3.83 – 3.68 (m, 12H), 2.90 (s, 4H).

^13^C NMR (101 MHz, CDCl_3_) δ 171.61, 167.40, 157.87, 149.75, 120.85, 119.72, 114.01, 110.57, 71.24, 27.17.

**Benzo-18-crown-6 modification of Gd-MSNs [9]:**

**Scheme S9.** Synthesis of benzo-18-crown-6 modified Gd-MSNs.

Gd-loaded MSNs (OH-Gd-MSNs, 75 mg, 0.3% amination) were dispersed, under sonication, in 5.625 mL of DMF. Then, 1.36 mg of benzo-18-crown-6 NHS ester and 150 μL of triethylamine were added to the reaction flask. The resultant mixture was stirred at r.t. for 24 h. After this, the nanoparticles were collected by centrifugation at 13,500 rpm for 20 min and washed three times with EtOH before leaving to dry under vacuum overnight.

**Synthesis of LnDO3AGlyPic (Ln = Eu, Gd):**

**Scheme S10.** Synthetic scheme to ligand DO3AGlyPic.

Compounds **[10]** and **[11]** were prepared according to literature procedures.^3, 4^

**Methyl 6-((2-(tert-butoxy)-2-oxoethyl)carbamoyl)picolinate [12]:**

**[11]** (1.000 g, 5.520 mmol) was stirred in thionyl chloride (8.21 g, 5.01 mL, 69.0 mmol) and 2 drops of DMF were added under nitrogen. The mixture was heated to 40 °C for 5 hours and the excess thionyl chloride removed under reduced pressure. The resulting off-white residue was further dried under vacuum overnight. The residue was then dissolved in 12.5 mL of dichloromethane and stirred at 0 °C under nitrogen. To the stirring solution, tert-butyl glycinate hydrochloride (925.4 mg, 5.520 mmol) mg of tert-butyl glycinate hydrochloride dissolved in 12.5 mL dichloromethane was added gradually, followed by dropwise addition of triethylamine (1.12 g, 1.54 mL, 11.0 mmol) of triethylamine. The mixture was then allowed to warm to room temperature and stirred for a further 3.5 hours. The solvent was then removed by rotary evaporation and the crude residue purified by column chromatography on silica using hexane and ethyl acetate (100:0 to 50:50). 732 mg (2.49 mmol, 45 %) of a colourless solid was obtained.

^1^H NMR (400 MHz, CDCl_3_) δ 8.48 (t, J = 5.8 Hz, 1H, **5**), 8.37-8.35 (dd, J = 7.8 Hz, 1.1 Hz, 1H, **8**), 8.24-8.22 (dd, J = 7.8 Hz, 1.1 Hz, 1H, **10**), 8.00 (t, J = 7.8 Hz, 1H, **9**), 4.18-4.16 (d, J = 5.8 Hz, 2H, **4**), 4.01 (s, 3H, **13**), 1.50 (s, 9H, **1**).

^13^C NMR (100 MHz, CDCl_3_) δ 168.7 (**3**), 165.1 (**12**), 163.8 (**6**), 149.8 (**7**), 146.9 (**11**), 138.6 (**9**), 127.5 (**10**), 125.6 (**8**), 82.4 (**2**), 53.0 (**13**), 42.2 (**4**), 28.2 (**1**).

HRMS (ESI+): calcd. for C_14_H_19_N_2_O_5_ [M+H]^+^ 295.1288, exp. 295.1292.

**Tert-butyl (6-hydroxymethyl)picolinoyl)glycinate** **[13]:**

**[12]** (732 mg, 2.49 mmol) was dissolved in 35 mL of methanol and cooled to 0 °C. To the stirring solution, sodium borohydride (333 mg, 8.80 mmol) was added in portions over 10 minutes. The solution was left to stir for a further 3 hours at 0 °C before 20 mL of saturated sodium bicarbonate solution was added and the methanol was then removed by rotary evaporation. The resulting aqueous solution was then extracted with chloroform. (5 × 50 mL). The organic layers were combined, dried with magnesium sulfate and evaporated to dryness. 558 mg (2.10 mmol, 84 %) of a colourless solid was obtained.

^1^H NMR (400 MHz, CDCl_3_) δ 8.36 (s, 1H, **5**), 8.10-8.08 (d, J = 7.7 Hz, 1H, **8**), 7.87-7.83 (t, J = 7.7 Hz, 1H, **9**), 7.48-7.46 (d, J = 7.7 Hz, 1H, **10**), 4.82 (s, 2H, **12**), 4.17-4.16 (d, J = 5.5 Hz, 2H, **4**), 1.50, (s, 9H, **1**).

^13^C NMR (100 MHz, CDCl_3_) δ 169.0 (**3**), 164.2 (**6**), 158.7 (**11**), 148.7 (**7**), 138.3 (**9**), 123.6 (**10**), 121.4 (**8**), 82.6 (**2**), 64.7 (**12**), 42.2 (**4**), 28.2 (**1**).

HRMS (ESI+): calcd. for C_13_H_19_N_2_O_4_ [M+H]^+^ 267.1339, exp. 267.1330.

**Tert-butyl (6-(chloromethyl)picolinoyl)glycinate** **[14]:**

**[13]** (558 mg, 2.21 mmol) was cooled to 0 °C under nitrogen. Thionyl chloride (1.96 g, 1.20 mL, 16.4 mmol) was added over the cooled solid and the reaction stirred for a further 1.5 hours. The excess thionyl chloride was removed under reduced pressure and the resulting residue taken up in 25 mL toluene then washed with 1 M NaHCO_3_ (3 × 15mL). The organic solution was then dried with magnesium sulfate and evaporated. 412 mg (1.45 mmol, 66 %) of a light brown solid was obtained and used without further purification.

^1^H NMR (400 MHz, CDCl_3_) 8.41 (s, 1H, **5**), 8.14 (dd, J = 7.8, 0.7 Hz, 1H, **8**), 7.89 (t, J = 7.8 Hz, 1H, **9**), 7.64 (dd, J = 7.8, 0.7 Hz, 1H, **10**), 4.70 (s, 2H, **12**), 4.16 (d, J = 5.5 Hz, **4**), 1.51 (s, 9H, **1**).

^13^C NMR (400 MHz, CDCl_3_) 169.0 (**3**), 164.0 (**6**), 155.6 (**11**), 149.1 (**7**), 138.7 (**9**), 125.7 (**10**), 121.8 (**8**), 82.5 (**2**), 46.2 (**12**), 42.2 (**4**), 28.2 (**1**).

HRMS (ESI+) calcd. for C_13_H_17_ClN_2_O_3_Na [M+Na]^+^ 307.0820, exp. 307.0805.

**tBuDO3A(tBuGlyPic)** **[15]:**

tri-tert-butyl 2,2',2''-(1,4,7,10-tetraazacyclododecane-1,4,7-triyl)triacetate hydrobromide (250 mg, 420 μmol) and sodium carbonate (224 mg, 2.12 mmol) were stirred in 7 mL of acetonitrile. To the stirring solution, **[14]** (150 mg, 527 μmol) in 7 mL acetonitrile was added. The mixture was then heated to reflux for 24 hours. The mixture was then filtered and solution evaporated to dryness. The crude product was purified by column chromatography on silica using dichloromethane and methanol (100:0 to 90:10). 275 mg (360 μmol, 85 %) of an off-white solid was obtained.

^1^H NMR (400 MHz, CD_3_CN) δ 8.80-8.77 (t, J = 6.0 Hz, 1H, **5**), 8.01-7.99 (dd, J = 7.7 Hz, 1.0 Hz, 1H, (**8**), 7.94-7.90 (t, J = 7.7 Hz, 1H, **9**), 7.59-7.57 (dd, J = 7.7 Hz, 1 Hz, 1H, **10**), 4.04-4.02 (d, J = 6.0 Hz, 2H, **4**), 3.71 (s(br), 2H, **12**), 3.20-2.10 (m, 20H, ring CH_2_),  1.47-1.41 (m, 36 H, tert-butyl CH_3_).

^13^C NMR (100 MHz, CD_3_CN) δ 174.3 (acetate C=O), 173.6 (acetate C=O), 169.7 (**3**), 165.7 (**6**), 158.0 (**11**), 151.1 (**7**), 139.4 (**9**), 128.9 (**10**), 121.8 (**8**), 83.4 (tert-butyl C), 83.0 (tert-butyl C), 82.1 (tert-butyl C), 60.3 (**12**), 57.4 (acetate CH_2_), 57.1 (acetate CH_2_), 50.6 (ring CH_2_), 43.3 (**4**), 28.3 (**1**, tert-butyl CH_3_).

HRMS (ESI+): calcd. for C_39_H_67_N_6_O_9_ [M+H]^+^ 763.4964, exp. 763.4986

**DO3AGlyPic:**

**6** (250 mg, 0.33 mmol) was dissolved in 2.5 mL dichloromethane and stirred. Trifluoroacetic acid (3.74 g, 2.50 mL, 33 mmol) was then added dropwise to the stirring mixture and left for a further 36 hours. The solvent was evaporated and then redissolved in 10 mL dichloromethane and re-evaporated. This was repeated once more with a further 10 mL of dichloromethane, followed by twice with 10 mL methanol. The resulting brown oil was taken up into 1 mL methanol and the product precipitated out by addition of diethyl ether. The white solid was isolated by centrifugation and dried under vacuum before being redissolved in methanol, reprecipitated with diethyl ether and centrifuged to yield 114 mg (0.21 mmol, 65 %) of a white solid.

^1^H NMR (400 MHz, D_2_O) δ 8.12-7.90 (m, 3H, pyridyl-H), 4.36-4.10 (m, 4H), 3.85-2.95 (m, 22H)

HRMS (ESI+) calcd. for C_23_H_35_N_6_O_9_ [M+H]^+^ 539.2460, exp. 539.2466.

**General procedure for complexation with LnOTf_3_:**

**Scheme S11.** Synthesis of LnDO3AGlyPic (Ln = Eu, Gd).

To DO3AGlyPic (1 Eq.) stirred in H_2_O:MeOH (1:1, 80 mM), LnOTf_3_ (1.25 Eq., Ln = Eu, Gd) dissolved in H_2_O:MeOH (1:1, 94 mM) was added and the reaction mixture heated to 50 °C. 1 M NaOH (3 Eq.) was added dropwise and the mixture left to stir for a further 48 hours. The resulting solution was filtered and the solvent removed. The resulting product was purified by dialysis resulting in off-white solid.

**EuDO3AGlyPic:**10.6 mg (19 μmol, 82 %).

^1^H NMR (400 MHz, D_2_O) δ 7.69, 7.26, 6.71, 6.21, 4.70, 3.68, 3.55, 3.48, 2.15, 1.51, 1.42, 1.22, -2.22, -3.17, -4.49, -6.75, -7.25, -9.43, -10.86, -12.54, -14.90.

HRMS (ESI+): calcd. for C_23_H_32_EuN_6_O_9_ [M+H]^+^ 689.1438, exp. 689.1434.

Luminescent lifetimes (λ_ex_ = 280 nm, λ_em_ = 615 nm): $\tau_{H_{2}O}=1.02 ms , \tau_{D_{2}O}=1.80 ms , q=0.21$.

**GdDO3AGlyPic:**
24.1 mg (35 μmol, 92 %).

HRMS (ESI+): calcd. for C_23_H_32_GdN_6_O_9_ [M+H]^+^ 694.1466, exp. 694.1464.

**Synthesis of LnDO3AGlyPic-MSN (q = 0 LnMSNs) (Ln = Eu, Gd):**

3.636 mg (5.25 µmol) of LnDO3AGlyPic (Ln = Eu, Gd) was dissolved in 2 mL of anhydrous DMF alongside 0.906 mg NHS and lowered to 0 °C under an argon atmosphere. 1.1 µL of diisopropylcarbodiimide (DIC) in 1 mL anhydrous DMF was injected dropwise. The mixture was stirred at 0 °C for 30 minutes, and allowed to rise to r.t. and stir for 45 minutes. 200 mg of NH_2_-MSNs were dried under vacuum at 50 °C then dispersed in 10 mL anhydrous DMF along with 190 µL of diisopropylethylamine (DIPEA). The activated ligand solution was added to the MSN suspension and the mixture was stirred at r.t. for 24 hours. The particles were collected by centrifugation. They were repeatedly washed and collected with DMF, DMF, EtOH, EtOH. The particles were dried under vacuum at 40 °C.

Luminescent lifetime of COOH-EuDO3AGlyPic-MSN (λ_ex_ = 280 nm, λ_em_ = 615 nm): $\boldsymbol{\tau}_{\boldsymbol{H}_{\boldsymbol{2}}\boldsymbol{O}}\boldsymbol{=0.95 ms ,}\boldsymbol{\tau}_{\boldsymbol{D}_{\boldsymbol{2}}\boldsymbol{O}}\boldsymbol{=1.78 ms , q=0.29}$.

Synthesis of dendritic mesoporous silica nanoparticles (native DMSNs)^5^:

Triethanolamine (TEOA, 400.0 mg) was added to ultrapure water (20 mL) under magnetic stirring at 80 °C for 30 min. Then, CTAB (304.0 mg) was added to the TEOA solution and stirred for a further 1 h. Ferrocenecarboxylic acid (91.2 mg) was added to the mixture and stirred for 3 h. TEOS (3.2 mL, 14.3 mmol) was subsequently added, and the solution stirred for 30 min. The particles were collected by centrifugation at 15 000 rpm for 20 min, with the precipitate washed several times with ethanol to remove any residual reactants to obtain the desired DMSNs. The surfactant templates were removed from the DMSNs by an extraction using a 10 vol% solution of hydrochloric acid in ethanol, under sonicating for 30 mins. This extraction process was repeated twice.

Synthesis of aminated dendritic mesoporous silica nanoparticles (NH_2_-DMSNs):

**10% NH_2_-DMSNs:** Native DMSNs (100 mg) were dispersed in a flask containing EtOH (10 mL) and H_2_O (5 mL), with APTES (39.0 µL) then added into the reaction mixture. The reaction was stirred at r.t. for 24 h, followed by the prior described ethanolic washing procedure (x3) and subsequent collection by centrifugation (15 000 rpm, 20 min). The particles were dried under vacuum overnight before further use.

**0.3% NH_2_-DMSNs:** Pristine DMSNs (100 mg) were dispersed in a flask containing EtOH (10 mL) and H_2_O (5mL), with APTES (1.17 µL) then added into the reaction mixture. The reaction was stirred at r.t. for 24 h, followed by an ethanolic washing procedure (x3) and subsequent centrifugal collection (15 000 rpm, 20 min). The particles were dried under vacuum overnight before further use.

Synthesis of Gd doped dendritic mesoporous silica nanoparticles (Gd-DMSNs):

50 mg of dried NH_2_-DMSNs (0.3% or 10% amination) were dispersed in 3.75 mL DMF, with 1,4,7,10-tetraazacyclododecane-1,4,7,10-tetraacetic acid mono-N-hydroxysuccinimide ester (DOTA-NHS ester, 1.90 mg, 2.5 μmol) and trimethylamine (37.5 μL, 0.27 mmol) then added to the DMSN dispersion. The reacting mixture was stirred for 24 h at room temperature, and collected by centrifugation (15000 rpm, 20 min) after three washing procedures with ethanol. The nanoparticles were re-dispersed in 2.5 mL EtOH, to which gadolinium (III) chloride hexahydrate (GdCl_3_∙6H_2_O, 1.86 mg, 5.0 μmol) was added, and the solution left stirring for 24 h at room temperature. The resultant particles were collected by centrifugation (15 000 rpm, 20 min) and subsequently washed three times with ethanol. The particles were dried under vacuum overnight, with the reaction of the 0.3% NH_2_-DMSNs named OH-Gd-DMSNs, and NH_2_-Gd-DMSNs from that with the 10% NH_2_-DMSNs.

Synthesis of Succinic anhydride modified Gd-DMSNs (COOH-Gd-DMSNs):

Dried NH_2_-Gd-DMSNs (25 mg) were dispersed in DMF (2.5 mL), followed by addition of succinic anhydride (37.5 mg). The reaction mixture was stirred at room temperature for 24 h, with the particles collected by centrifugation (15 000 rpm, 20 min) and washed twice with DMF (x2) and once with EtOH (x1). The particles were dried under vacuum overnight prior to further use.

EDTA Dialysis on kosmotrope-modified Gd-MSNs:

COOH-Gd-MSNs (10 mg) were dispersed in 3.0 mL of H_2_O. The nanoparticle dispersion was placed in a 5 mL dialysis bag (Spectra/Por®, molecular cut-off: 500 – 1000 Da) and was gently stirred in a 4 L solution of 1 mM ethylenediaminetetraacetic acid trisodium salt hydrate. The EDTA solution was replaced at 4 h, 22 h, and 30 h intervals with fresh 1 mM EDTA solution (4 L). After 50 h, the nanoparticles were collected by centrifugation at 13500 rpm for 20 min. The pellet was washed twice with H_2_O, once with EtOH, and then dried under vacuum overnight. The relaxivities of these particles were then measured using a 1.4 T NMR spectrometer.

**Results and discussion**

**Functionalised MSNs**


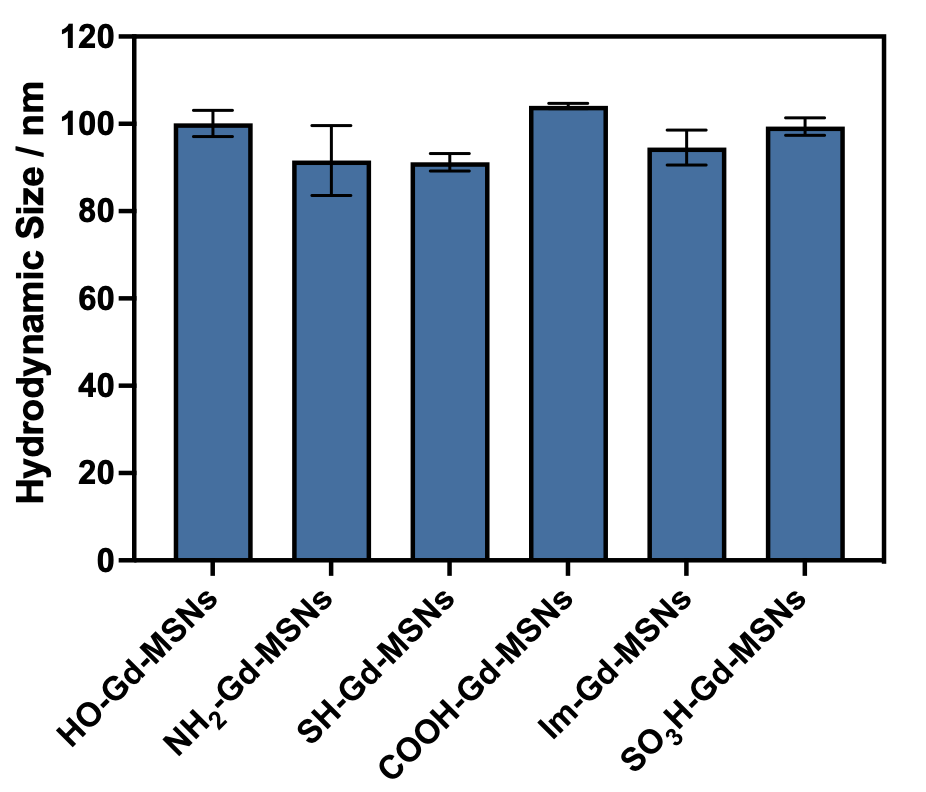


**Figure S1.** DLS data for a 1 mg mL^-1^ aqueous solution of the desired functionalised MSN in order to highlight no appreciable changes in hydrodynamic size across all particle formulations.

| Sample | Hydrodynamic size / nm | TEM resolved size / nm |
| --- | --- | --- |
| OH-Gd-MSNs | 100.1 ± 3.0 | 54.9 ± 7.0 |
| NH_2_-Gd-MSNs | 91.6 ± 8.0 | 51.9 ± 4.0 |
| SH-Gd-MSNs | 91.2 ± 2.0 | 67.2 ± 3.0 |
| COOH-Gd-MSNs | 104.1 ± 0.6 | 62.7 ± 3.0 |
| Im-Gd-MSNs | 94.6 ± 4.0 | - |
| SO_3_H-Gd-MSNs | 99.4 ± 2.0 | 56.6 ± 4.0 |

**Figure S2.** A table summarising the both the hydrodynamic size recorded by DLS and TEM resolved size for the MSN particle formulations included in this work.

**Figure S3.** Barrett-Joyner-Halenda (BJH) derived adsorption/desorption measurements to elucidate the pore diameter for the initial parent MSN architectures. Each particle, along with their associated pore size, is annotated above with an observed pore diameter of 2.6 nm for the SH-MSNs, 3.2 nm for the OH-MSNs and 3.5 nm for the NH_2_-MSNs.

**Amino Anchor Groups Estimation**

Molar mass of TEOS/APTES = 208.33 / 221.3 g mol^-1^

Molar ratio of TEOS:APTES in 0.3 APTES mol% MSN = $\frac{208.33*99.7}{221.3*0.3}$ = 312.855:1

Mass % of APTES per MSN = $\frac{1}{312.855+1}*100$ = 0.318%

Mass of MSN = $\frac{4}{3}\pi*r^{3}*\rho=\frac{4}{3}\pi*\left( 30 \mathrm{nm} \right)^{3}*2 g cm^{-1}$ = 2.262 x 10^-16^ g

Number of APTES per MSN = 2.262 x 10^-16^ g * 0.00318 * (1/221.3) * *N*_A_ = 1961.2

| Sample | Gd^3+^/ MSN |
| --- | --- |
| OH-Gd-MSNs | 2028 |
| NH_2_-Gd-MSNs | 2693 |
| SH-Gd-MSNs | 2483 |
| COOH-Gd-MSNs | 1807 |
| Im-Gd-MSNs | 1099 |
| SO_3_H-Gd-MSNs | 829 |

**Figure S4.** A table summarising the number of estimated chelated Gd^3+^ atoms per MSN for different formulations.

**
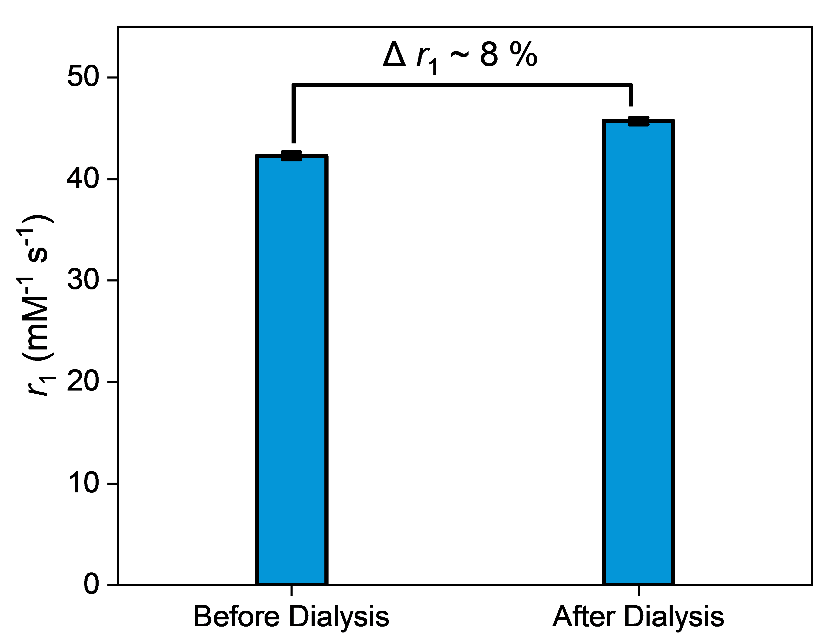
**

**Figure S5.** Longitudinal relaxivity values for the COOH-Gd-MSNs before and after EDTA (1 mM, 4 L) dialysis, with an insignificant change in $r_{1}$ ($\Delta r_{1}$ $\sim$ 8 %) observed.


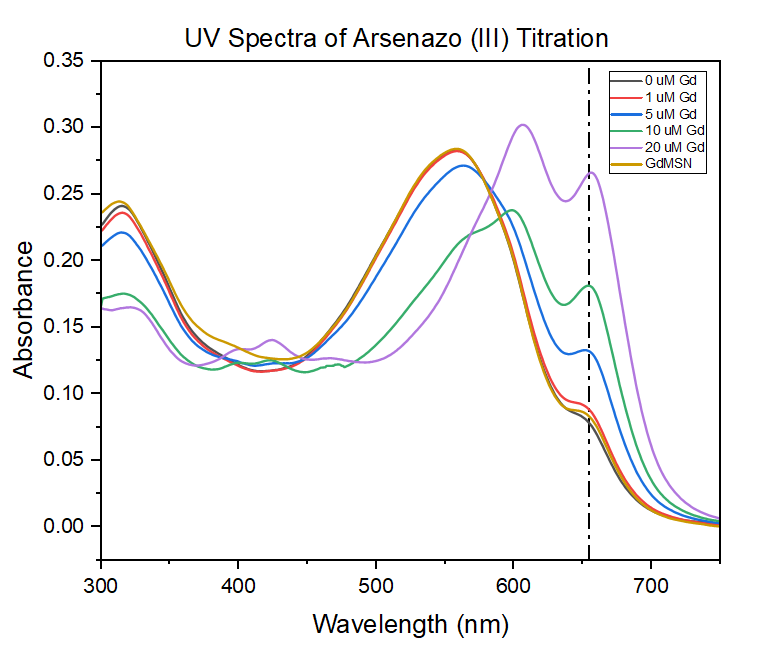


**Figure S6.** Determination of the concentration of unchelated Gd(III) present within the native Gd-MSNs. Analysis of the titration revealed that there was 0.1765 μM of free Gd(III) present at a concentration of 1 mg mL^-1^ of particles (0.005% of total Gd(III)).


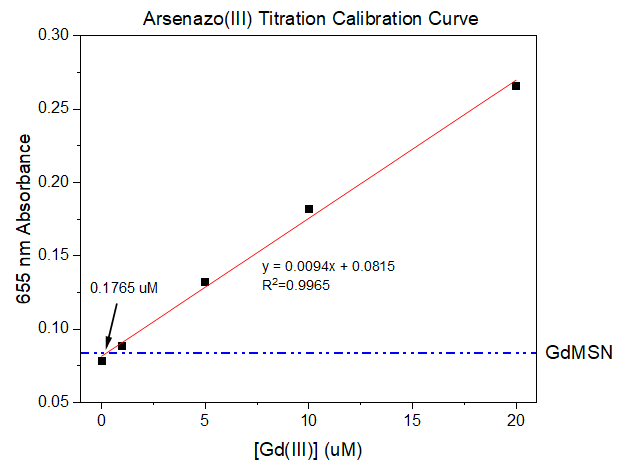


**Figure S7.** Determination of the concentration of unchelated Gd(III) present within the native Gd-MSNs. A calibration curve of various Gd(III) concentrations using the UV-vis absorbance of the lanthanide sensitive dye Arsenazo(III) at 655 nm. Analysis of the titration revealed that there was 0.1765 μM of free Gd(III) present at a concentration of 1 mg mL^-1^ of particles (0.005% of total Gd(III)).


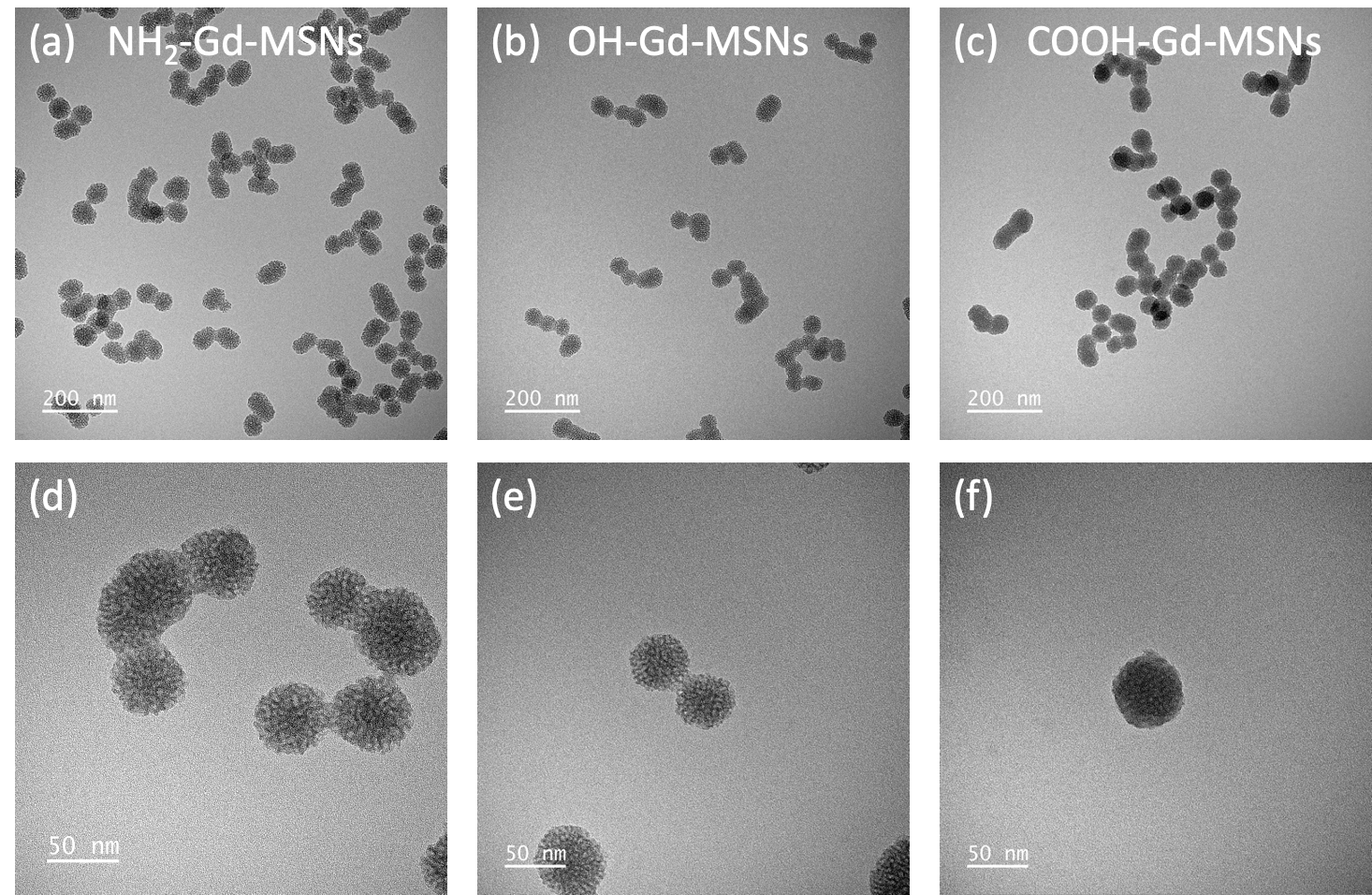


**Figure S8.** TEM images for the functionalised MSNs, clearly highlighting both their spherical and mesoporous morphology, and high colloidal stability.

**Figure S9.** DLS data, reporting both the hydrodynamic size and ζ-potential measurements, for a 1 mg mL^-1^ aqueous solution of the desired functionalised particle across a full pH range. (a) NH_2_-Gd-MSNs, (b) SH-Gd-MSNs, (c) COOH-Gd-MSNs, (d) SO_3_H-Gd-MSNs and (e) Im-Gd-MSNs.

**Figure S10.** ATR-IR spectra for the modified MSN architectures included in this work, highlighting the Si-O-Si stretch corresponding to the underlying silica architecture and clear removal of the templating surfactant.

**Relaxivity Values**

**
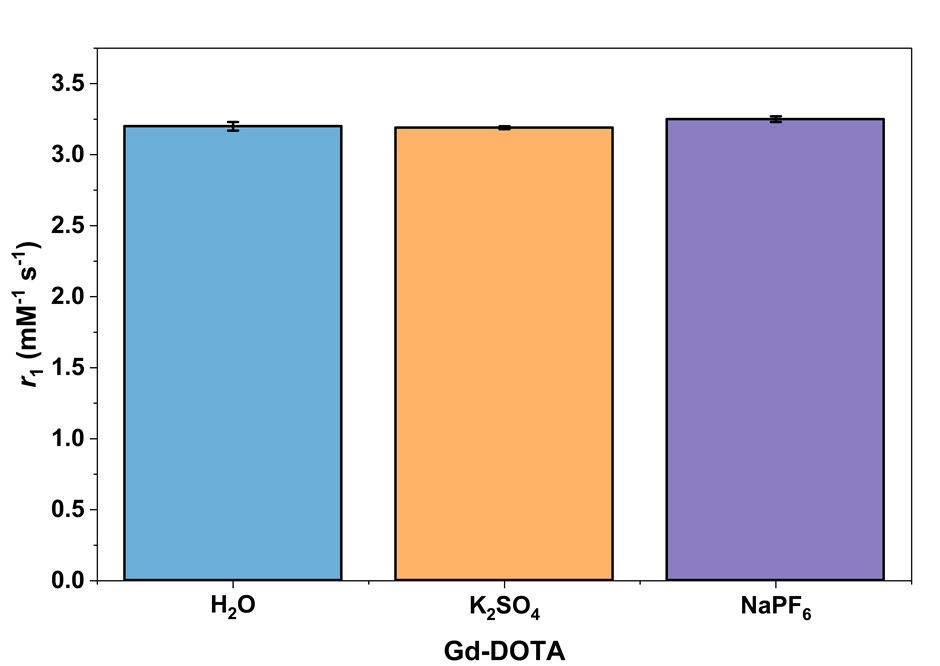
**

**Figure S11.** Longitudinal relaxivity values for Gd-DOTA in the presence of a kosmotropic ion (5 mM SO_4_^2-^) or a chaotropic ion (10 mM PF_6_^-^). As expected, $r_{1}$ is unchanged given the lack of a particle interface capable of ordering associated viscous water.





**Figure S12.** Longitudinal relaxivity values (at 1.4 T) recorded for a 1 mg mL^-1^ (pH 7.4) dispersion of amine or carboxylate modified MSNs. The integrated paramagnetic chelate was either the native Gd-DOTA or q = 0 GdDO3AGlyPic ligand, included side by side to enable a comparison between the two. As can be observed, high relaxivities remain even when modified with the q = 0 ligand, indicative of a dominant OS relaxation pathway. Relaxivities can only be “switched off” on addition of a water structure disrupting chaotropic ion (PF_6_^-^).

**Mathematical modelling**

The observed longitudinal relaxivity value, denoted $r_{1}$, quantifies the ability of an MRI contrast agent to shorten the $T_{1}$ relaxation time of water.^6, 7^ It can be separated into the sum of its inner-sphere (IS), second-sphere (SS) and outer-spere contribution (OS) (Eq. 1):

$r_{1}=r_{1}^{IS}+r_{1}^{SS}+ r_{1}^{OS}$ (1)

It has been prior reported that, for Gd-DOTA the IS component accounts for approximately 50%, the SS component for approximately 10% (often considered negligible)^6^ and the OS component for the remaining 40% (summarised in ESI 12).^8, 9^ The OS contribution is, then, both notable and expected to be even more significant for MSN-based agents. Herein we have sought to highlight a controlled modulation of this OS contribution through the integration of strong water ordering groups.^8, 10, 11^


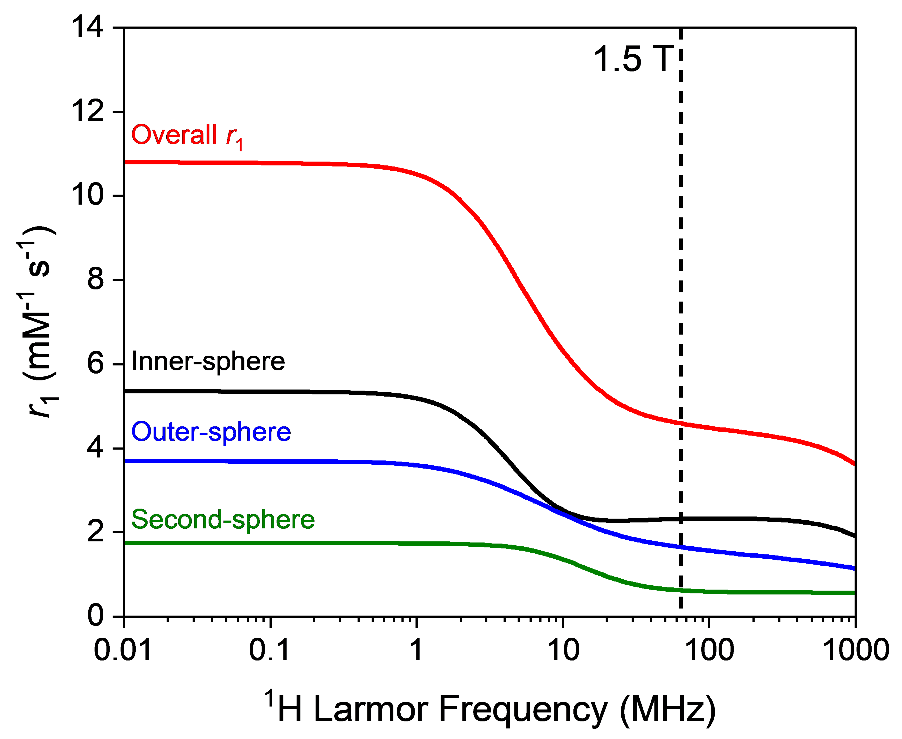


**Figure S13.** A simulated NMRD plot summarising the different contributions to the overall $r_{1}$ value (red), specifically the IS (black), OS (blue) and SS (green) components. The parameters for a typical molecular Gd-DOTA chelate are estimated at $r_{GdH}^{IS}$ = 0.31 nm; $r_{GdH}^{SS}$ = 0.35 nm; $r_{GdH}^{OS}$ = 0.40 nm; $\tau_{M}^{IS}$= 244 ns; $\tau_{M}^{SS}$= 25 ps; $\tau_{R}$ = 77 ps; $\tau_{D}$ = 62 ps; $q$ = 1; $q'$ = 2; $\Delta^{2}$ = $3.8\times{10}^{19} s^{-2}$; $\tau_{v}$ = 11 ps. This figure highlights the significant contribution ($\sim$ 40 %) from the outer-sphere contribution to the overall relaxivity.

**Inner-sphere contribution:**

The expression for $r_{1}^{IS}$ is described in equation (2):

| $r_{1}^{IS}=q[H_{2}O]/(T_{1M}+\tau_{M})$ | (2) |
| --- | --- |

where $q$ is the number of coordinated water molecules bound to the paramagnetic ion ($q=1$ set in this case); $[H_{2}O]$ is the water concentration; $T_{1M}$ is the relaxation time of the water bound to the ion; $\tau_{M}$ is the residence lifetime of the inner-sphere water molecules.

The reciprocal of $T_{1M}$ can be expressed as the sum of the dipolar coupling relaxation rates, ${1/T}_{1}^{\mathrm{DD}}$, the scalar interaction relaxation rate, ${1/T}_{1}^{\mathrm{SC}}$, and the Curie spin rate, ${1/T}_{1}^{\mathrm{CS}}$. The scalar-interaction and Curie-spin terms are often deemed to be negligible.^6^ As a result, ${1/T}_{1M}$ is often written as the the dipole-dipole relaxation rate, ${1/T}_{1}^{\mathrm{DD}}$.

| $\frac{1}{T_{1M}}=\frac{1}{T_{1,DD}}+\frac{1}{T_{1,SC}}+\frac{1}{T_{1,CS}}\approx\frac{2}{15}\frac{C}{r_{\mathrm{GdH}}^{6}}\left[ \frac{F^{2}3\tau_{cG1}}{1+\omega_{I}^{2}\tau_{cG1}^{2}}+\frac{(1-F^{2})3\tau_{cL1}}{1+\omega_{I}^{2}\tau_{cL1}^{2}} \right]$ | (3) |
| --- | --- |
| $\frac{1}{\tau_{cG1}}=\frac{1}{\tau_{\mathrm{RG}}}+\frac{1}{\tau_{M}}+\frac{1}{T_{1e}}$ | (4) |
| $\frac{1}{\tau_{cL1}}=\frac{1}{\tau_{cG1}}+\frac{1}{\tau_{\mathrm{RL}}}$ | (5) |

where the constant $C$ is $\gamma_{I}^{2}g^{2}\mu_{B}^{2}\left( \frac{\mu_{0}}{4\pi} \right)^{2}S\left( S+1 \right)$; $\gamma_{I}$ is the proton gyromagnetic constant ($\gamma_{I}=2.675\times{10}^{8} T^{-1} s^{-1}$); $g$ is the electronic g-factor ($g=2$ for Gd^3+^); $S$ is the total electron spin of the paramagnetic ion ($S= 7/2$ for Gd^3+^); $\mu_{B}$ is the Bohr magneton ($\mu_{B}=9.274\times{10}^{-24} J T^{-1}$); $\mu_{0}$ is the vacuum permeability ($\mu_{0}=1.257\times{10}^{-6} N A^{-1}$); $r_{\mathrm{GdH}}$ is the distance between the metal ion and the bound water proton ($r_{\mathrm{GdH}}$ set as $0.31 \mathrm{nm}$);^6^ $\omega_{H}$ and $\omega_{S}$ are the angular proton and electronic Larmor frequencies (with $\omega_{S}=658\omega_{I}$, $\omega_{I}=\gamma_{I}B$, with $B$ the magnetic field strength); $F^{2}$ denotes an order parameter that takes a value between 0 and 1; $\tau_{\mathrm{RG}}$ is the global correlation time; $\tau_{\mathrm{RL}}$ is the local correlation time; $\tau_{cL1}$ is the local correlation time which accounts for fast local motion ($\tau_{\mathrm{RL}}$).

$T_{ie}$ ($i=1,2$) are given by equations (6) and (7):

| $\frac{1}{T_{1e}}=\frac{1}{25}\Delta^{2}\tau_{v}\left[ 4S\left( S+1 \right)-3 \right]\left[ \frac{1}{1+\omega_{s}^{2}\tau_{v}^{2}}+\frac{4}{1+{4\omega}_{s}^{2}\tau_{v}^{2}} \right]$ | (6) |
| --- | --- |
| $\frac{1}{T_{2e}}=\frac{1}{25}\Delta^{2}\tau_{v}\left[ 4S\left( S+1 \right)-3 \right]\left[ \frac{5}{1+\omega_{s}^{2}\tau_{v}^{2}}+\frac{2}{1+{4\omega}_{s}^{2}\tau_{v}^{2}}+3 \right]$ | (7) |

where $\Delta^{2}$ is the mean square zero field splitting (ZFS) energy and $\tau_{v}$ is the correlation time for splitting.

In equations (2) and (3), $\tau_{M}$ can be estimated at 250 – 710 ns according to works by Botta and co-workers.^12-14^ In equations (4) and (5), the internal motion (*i.e.*, internal flexibility of the anchored Gd complex) is highlighted, which has been described by the model proposed by Lipari and Szabo.^15^ Depending on the value of $F^{2}$ the motion is either governed by the global relaxation time ($F^{2}=1$) or the fast local motion ($F^{2}=0$) of the complex. In this work, we employ the values of Gd-MSNs from those of a similar chemical structure ($F^{2}=0.5$ and $\tau_{\mathrm{RL}}=2.4$ ns) as described in previous work.^12^ In equations (6) and (7), $\Delta^{2}$ can be estimated as $3.8\times{10}^{19} s^{-2}$ and $\tau_{v}$ is set at $11$ ps based on the literature values.^16^ Using these parameters, the overall inner-sphere relaxivities can be calculated as 15.1 – 24.5 mM^-1^ s^-1^ at 1.4 T, which are significantly smaller than any of the kosmotrope-modified Gd-MSNs. As a result, it is reasonable to consider the presence of an additional composite OS contribution (with more analysis below).

**Outer-sphere contribution:**

The OS contribution can be summarised by the following set of equations:

$r_{1}^{OS}=\frac{1}{c_{Gd}}\left( \frac{1}{T_{1}} \right)_{OS}=\left( \frac{32\pi}{405} \right)C_{DD}\frac{N_{A}}{aD}Re\left[ 3j\left( \omega_{I} \right)+7j{(\omega}_{S}) \right]$ (2)

$c_{\mathrm{Gd}}$ is the concentration (in mM) of the metal ions in solution, $a$ the distance between the metal ion and proton (estimated at $0.5 \mathrm{nm}$),$D$ the sum of the diffusion coefficients of bulk water and the complex, and $N_{A}$ Avogadro’s constant ($N_{A}=6.023\times{10}^{23}$). $C_{\mathrm{DD}}$ is given by:

$C_{\mathrm{DD}}=\gamma_{I}^{2}g^{2}\mu_{B}^{2}\left( \frac{\mu_{0}}{4\pi} \right)^{2}S\left( S+1 \right)$ (3)

with $\gamma_{I}$ the proton gyromagnetic constant ($\gamma_{I}=2.675\times{10}^{8} T^{-1} s^{-1}$), $g$ the electronic g-factor ($g=2$), $S$ the total electron spin of the material ion ($S= 7/2$ for Gd^3+^), $\mu_{B}$ the Bohr magneton ($\mu_{B}=9.274\times{10}^{-24} J T^{-1}$), $\mu_{0}$ the vacuum permeability ($\mu_{0}=1.257\times{10}^{-6} N A^{-1}$). $\omega_{I}$ and $\omega_{S}$ are the angular proton and electronic Larmor frequencies (with $\omega_{S}=658\omega_{I}$, $\omega_{I}=\gamma_{I}B$, with $B$ the magnetic field strength), and $A$ the hyperfine coupling constant (in J). $\mathrm{Re}$ is the real part of the spectral density function $j(\omega$) given by:

$j\left( \omega\right)=\left[ 4+\left( i\omega\tau_{D}+\frac{\tau_{D}}{T_{1e}} \right)^{1/2} \right]/\left[ 4+4\left( i\omega\tau_{D}+\frac{\tau_{D}}{T_{1e}} \right)^{1/2}+\frac{16}{9}\left( i\omega\tau_{D}+\frac{\tau_{D}}{T_{1e}} \right)+\frac{4}{9}\left( i\omega\tau_{D}+\frac{\tau_{D}}{T_{1e}} \right)^{3/2} \right]$ (4)

with $\tau_{D}$ representing the diffusion correlation time and summarised by:

$\tau_{D}={a^{2}}/D$ (5)

$T_{ie}$ is given by:

$\frac{1}{T_{1e}}=\frac{1}{25}\Delta^{2}\tau_{v}\left[ 4S\left( S+1 \right)-3 \right]\left[ \frac{1}{1+\omega_{s}^{2}\tau_{v}^{2}}+\frac{4}{1+{4\omega}_{s}^{2}\tau_{v}^{2}} \right]$ (6)

where $\Delta^{2}$(estimated at $3\times{10}^{19} s^{-2}$) is the mean square zero field splitting (ZFS) energy and $\tau_{v}$ (estimated at $15 \mathrm{ps}$) is the correlation time for splitting.

$\boldsymbol{q}$ **value measurements**

It should be noted that the resolved differences in $q$ are initially surprising given that the same chelate and chemical modification procedure was employed for all particle formulations and that ICP-MS measurements and representative EDTA dialysis confirmed the absence of any physisorbed Gd^3+^. It has, however, been previously reported that the rate constants for the depopulation of excited states can be influenced by the quenching effect of closely diffusing outer-sphere water molecules.^1^ These closely diffusing water molecules can enable vibrational energy transfer over long distances (up to 5 Å, *i.e.*, greater than those associated with typical IS and SS processes), affecting the associated resolved $q$ value of the lanthanide-chelate.^1^ A single such water has been shown to contribute $\sim$ 20 % to a calculated molecular $q$ value; scaling this to the presence of a reasonable 2 - 4 associated water molecules within the confines of a kosmotrope modified MSN channel thus reasonably generates the numbers resolved herein.^17^ Regardless of the influence of local water on experimentally resolved $q$ values, it is important to note that these resolved $q$ differences between particle formulations cannot account for the relaxivity differences; *e.g.*, an increase in $q$ from 1.1 for NH_2_-Gd-MSNs, to 2.1 for OH-Gd-MSNs would generate a change in $r_{1}$ of $\sim$ 90% by SBM theory *i.e.* much less than the 250% increase in $r_{1}$ between these two samples. We propose then that the resolved $q$ values have a realistic basis but that they are also not a dominant part of the effects that we resolve (which we ascribe to differences in *D*, *i.e.*, affecting the OS mechanism).


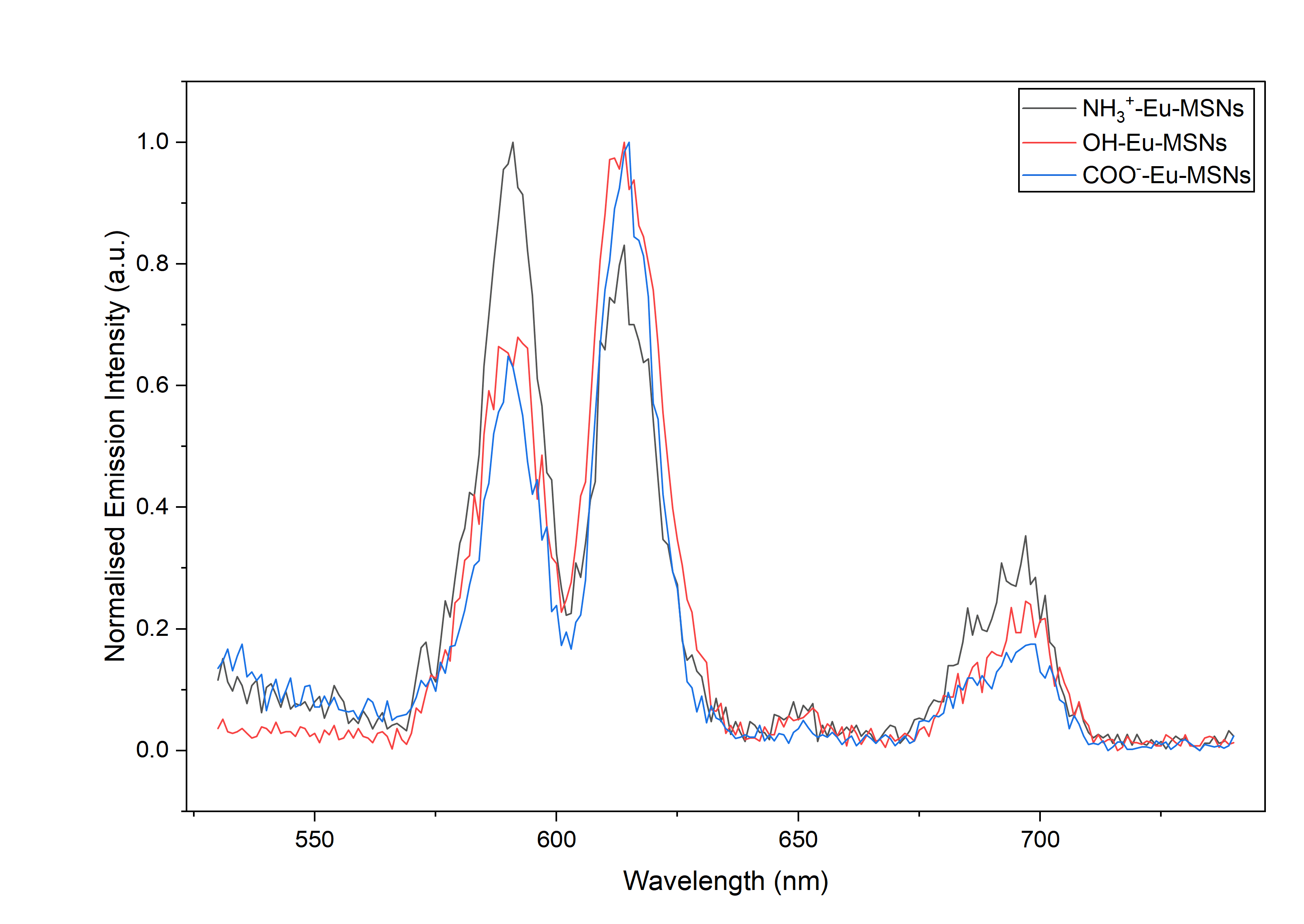


**Figure S14.** Time-gated emission spectra (λ_ex_ = 393 nm, uncorrected) of the NH_3_^+^-Eu-MSNs, OH-Eu-MSNs, and COO^-^-Eu-MSNs. It should be noted that due to colloidal stability issues at high particle concentrations, the NH_3_^+^-Eu-MSNs were measured in acetate buffer at pH 4.0, whereas the OH- and COO^-^ -Eu-MSNs were recorded in HEPES buffer at pH 7.0.

| Sample | $\boldsymbol{\tau}_{\mathbf{H}_{\mathbf{2}}\mathbf{O}}$ / ms | $\boldsymbol{\tau}_{\mathbf{D}_{\mathbf{2}}\mathbf{O}}$ / ms | $\boldsymbol{q}$ |
| --- | --- | --- | --- |
| NH_3_^+^-Eu-MSNs | 0.55 | 1.77 | 1.1 |
| OH-Eu-MSNs | 0.40 | 2.37 | 2.1 |
| COO^-^-Eu-MSNs | 0.43 | 1.93 | 1.8 |

**Figure S15.** A table summarising the rate constants for the depopulation of excited states for the integrated Eu-chelate within the chemically modified Eu-doped MSNs samples. These were recorded in H_2_O and D_2_O buffered solutions at pH 4.0 and 7.0. Where λ_ex_ = 393 nm and λ_em_ = 615 nm. The corresponding $q$ value is calculated using the above equation. All luminescent lifetimes are ± 10%.

***q* = 0 analysis**

| Sample | $\boldsymbol{\tau}_{\mathbf{H}_{\mathbf{2}}\mathbf{O}}$ / ms | $\boldsymbol{\tau}_{\mathbf{D}_{\mathbf{2}}\mathbf{O}}$ / ms | $\boldsymbol{q}$ |
| --- | --- | --- | --- |
| EuDO3AGlyPic | 1.02 | 1.80 | 0.21 |
| COO^-^-EuDO3AGlyPic-MSNs | 0.95 | 1.78 | 0.29 |

**Figure S16.** A table summarising the rate constants for the depopulation of excited states for the integrated Eu-chelate within the carboxylate modified EuDO3AGlyPic-MSNs, recorded in H_2_O and D_2_O at pH 7.0. Where λ_ex_ = 280 nm and λ_em_ = 615 nm. The corresponding $q$ value is calculated using the above equation. All luminescent lifetimes are ± 10%.


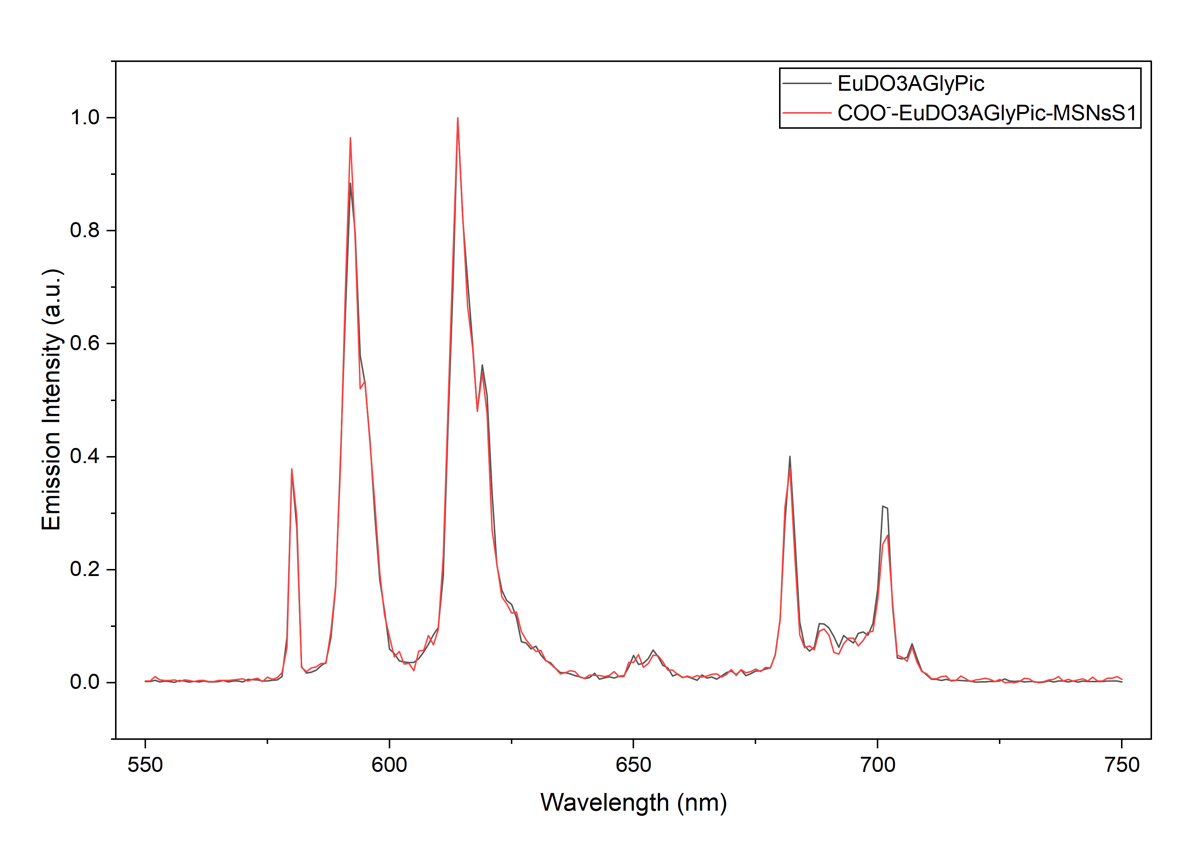


**Figure S17.** Time-gated emission spectra (λ_ex_ = 280 nm, uncorrected) of the discrete EuDO3AGlyPic chelate and carboxylate-modified EuDO3AGlyPic-MSNs. No change in spectral profile indicates consistent coordination environments in the discrete and MSN incorporated material.

**MSN MRI Analysis**

**
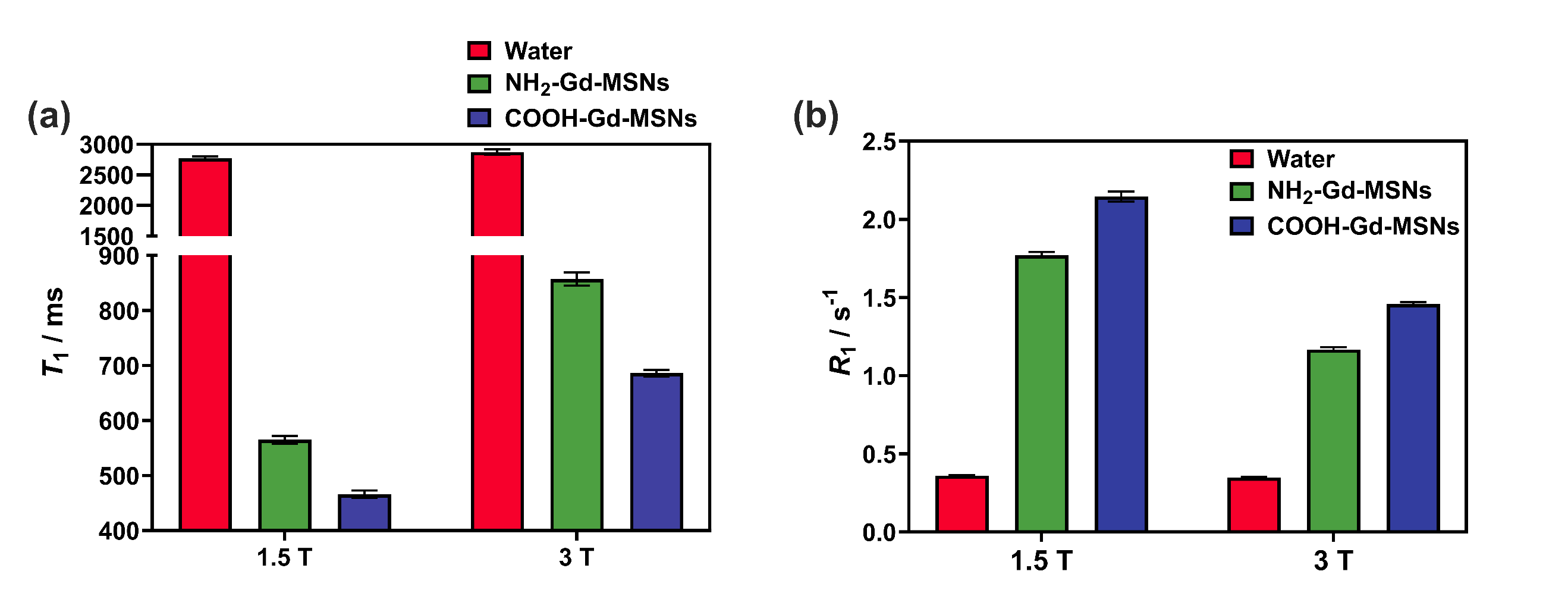
**

**Figure S18.** (a) MRI derived longitudinal relaxation times recorded at both 1.5 T and 3 T for the NH_2_-Gd-MSNs and the COOH-Gd-MSNs. (b) The corresponding relaxation rate measurements for the aminated and carboxylate MSNs. The samples were prepared by dispersing the particles (keeping the Gd-concentration at 0.04 mM) in water.


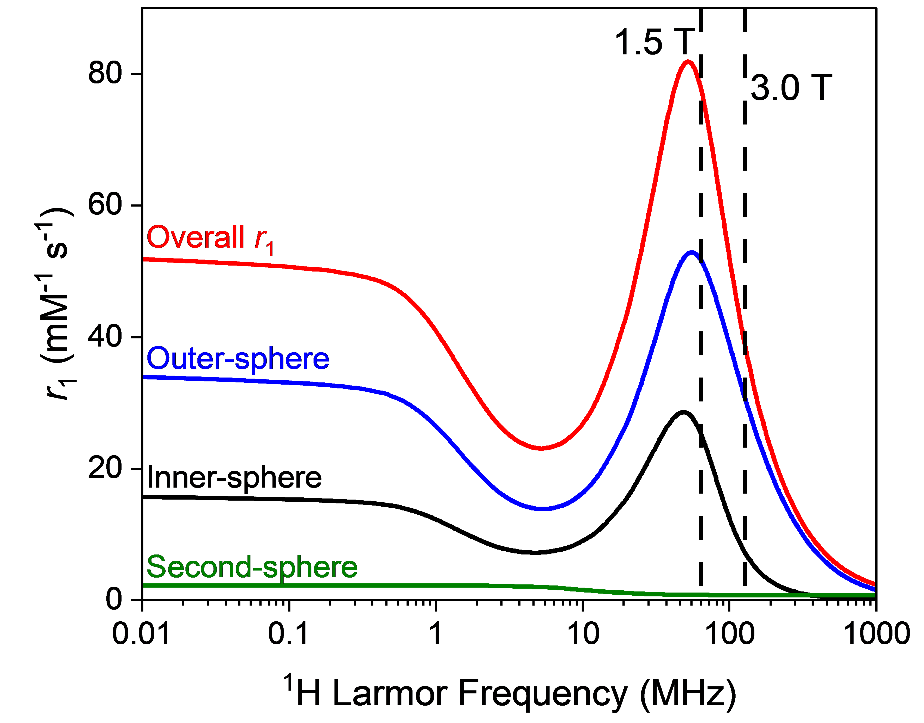


**Figure S19.** The effect of varying the applied external magnetic field on the theoretical relaxivity value, as derived by SBM theory. Here, the Gd-DOTA structure was modelled with an extended/optimized diffusional correlation time ($\tau_{D}$ = 6 ns). The other parameters used were: $r_{GdH}^{IS}$ = 0.31 nm; $r_{GdH}^{SS}$ = 0.35 nm; $r_{GdH}^{OS}$ = 0.40 nm; $\tau_{M}^{IS}$= 244 ns; $\tau_{M}^{SS}$= 25 ps; $\tau_{R}$ = 77 ps; $q$ = 1; $q'$ = 2; $\Delta^{2}$ = $3.8\times{10}^{19} s^{-2}$; $\tau_{v}$ = 11 ps. This modelled NMRD plot highlights the significantly enhanced $r_{1}$ solely due to a change in $\tau_{D}$. Moreover, this Figure serves as a reference, highlighting the decrease in $r_{1}$ for all of the individual components as field increases from 1.5 T to 3 T.

**Dendritic (larger pore aperture) MSNs results and discussion**

**Characterisation**

**Figure S20.** ATR-IR spectra for NH_2_-Gd-DMSNs (green), OH-Gd-DMSNs (light blue) and COOH-Gd-MSNs (dark blue) with the underlying silica architectural peak annotated.

**
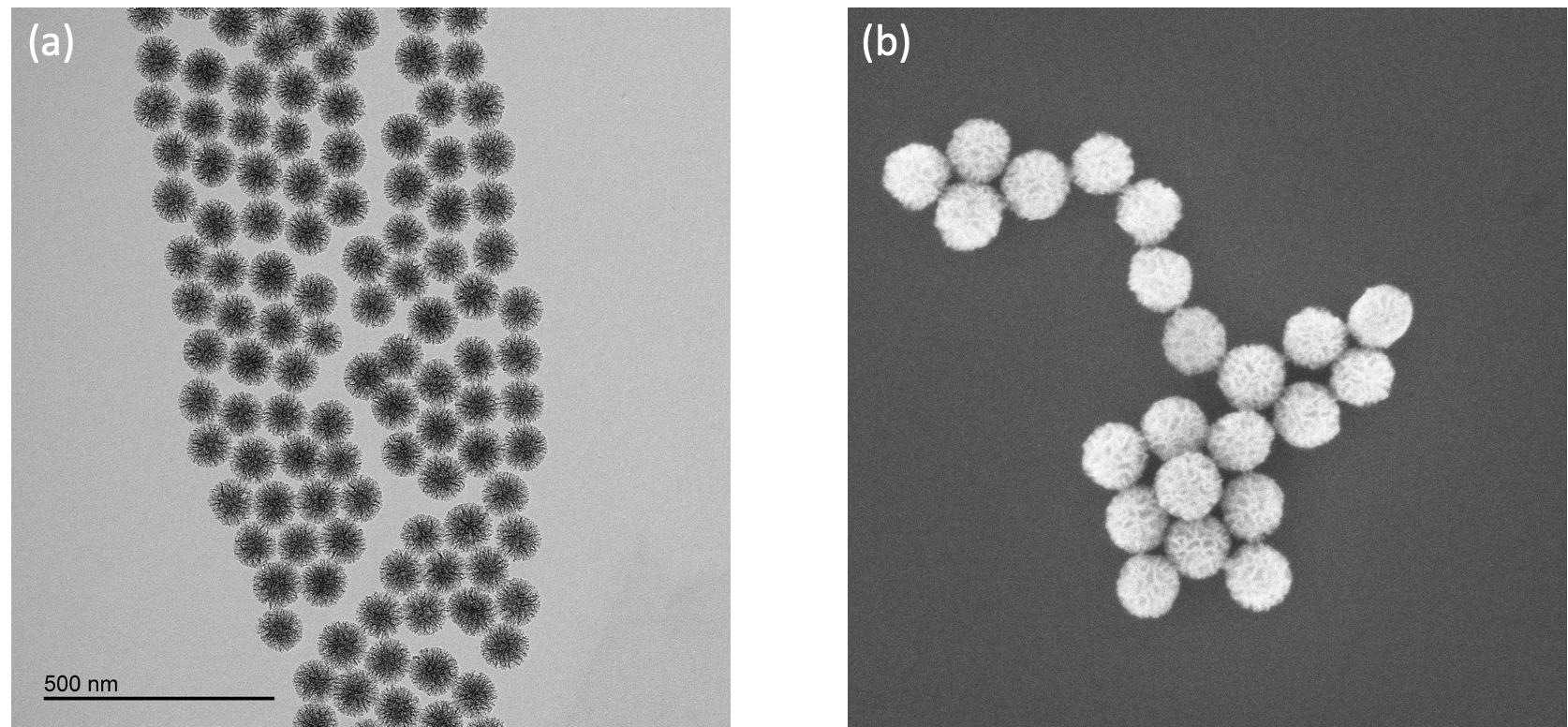
**

**Figure S21.** (a) TEM images of the DMSNs. (b) SEM images of the DMSNs.

**Figure S22.** Hydrodynamic sizes of all DMSN formulations employed in this work, as recorded for a 1 mg mL^-1^ solution of the particles by DLS.

**Figure S23.** BJH measurements to elucidate the pore diameter for the NH_2_-Gd-DMSNs, OH-Gd-DMSNs and COOH-Gd-DMSNs.

***q* value measurements for the dendritic (larger pore) MSNs**

| Sample | $\boldsymbol{\tau}_{\mathbf{H}_{\mathbf{2}}\mathbf{O}}$ | $\boldsymbol{\tau}_{\mathbf{D}_{\mathbf{2}}\mathbf{O}}$ | $\boldsymbol{q}$ |
| --- | --- | --- | --- |
| NH_2_-Eu-DMSNs | 0.57 | 1.57 | 0.9 |
| OH-Eu-DMSNs | 0.49 | 2.25 | 1.6 |
| COOH-Eu-DMSNs | 0.38 | 1.19 | 1.8 |

**Figure S24.** A table summarising the rate constants for the depopulation of excited states for the integrated Eu-chelate within the chemically modified Eu-doped DMSNs samples. These were recorded in H_2_O and D_2_O buffered solutions at pH 4.0 and 7.0, where λ_ex_ = 393 nm and λ_em_ = 615 nm. All luminescent lifetimes are ± 10%.

**Relaxivity measurements for the dendritic (larger pore) MSNs**

**
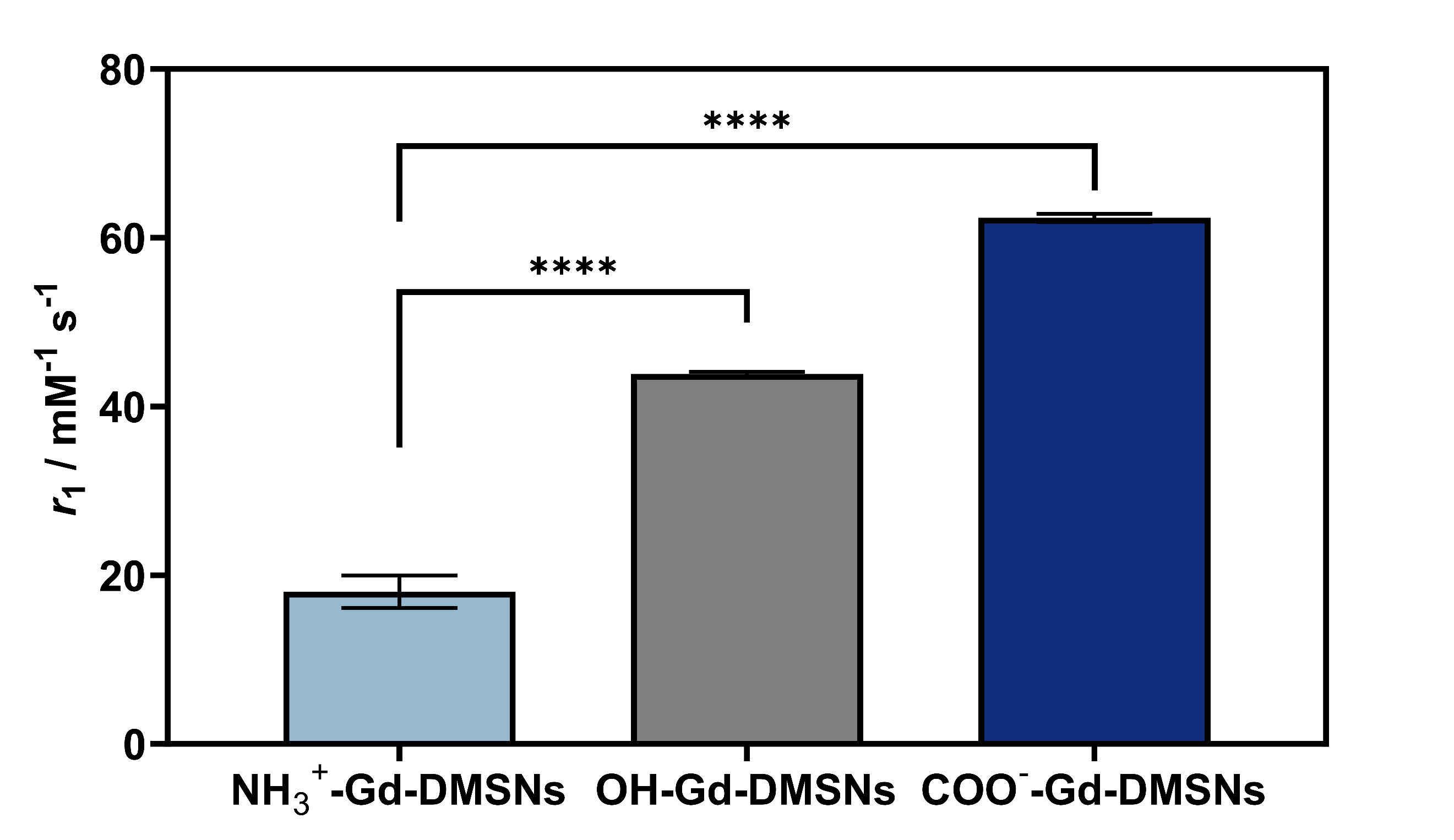
**

**Figure S25.** Longitudinal relaxivity measurements recorded by NMR (1.4 T) for the DMSNs at pH 7.0.

**DMSNs MRI measurements**

**Figure S26.** MRI derived (a) longitudinal relaxation times recorded by a clinical MRI scanner at both 1.5 T and 3 T for all DMSN formulations. (b) Longitudinal relaxation rates for the DMSNs, as recorded by the MRI scanner, at 1.5 T and 3 T.

**Figure S27.** The MRI derived longitudinal relaxivities for the NH_2_-Gd-DMSNs, OH-Gd-DMSNs and COOH-Gd-DMSNs recorded at (a) 1.5 T and (b) 3 T.

**
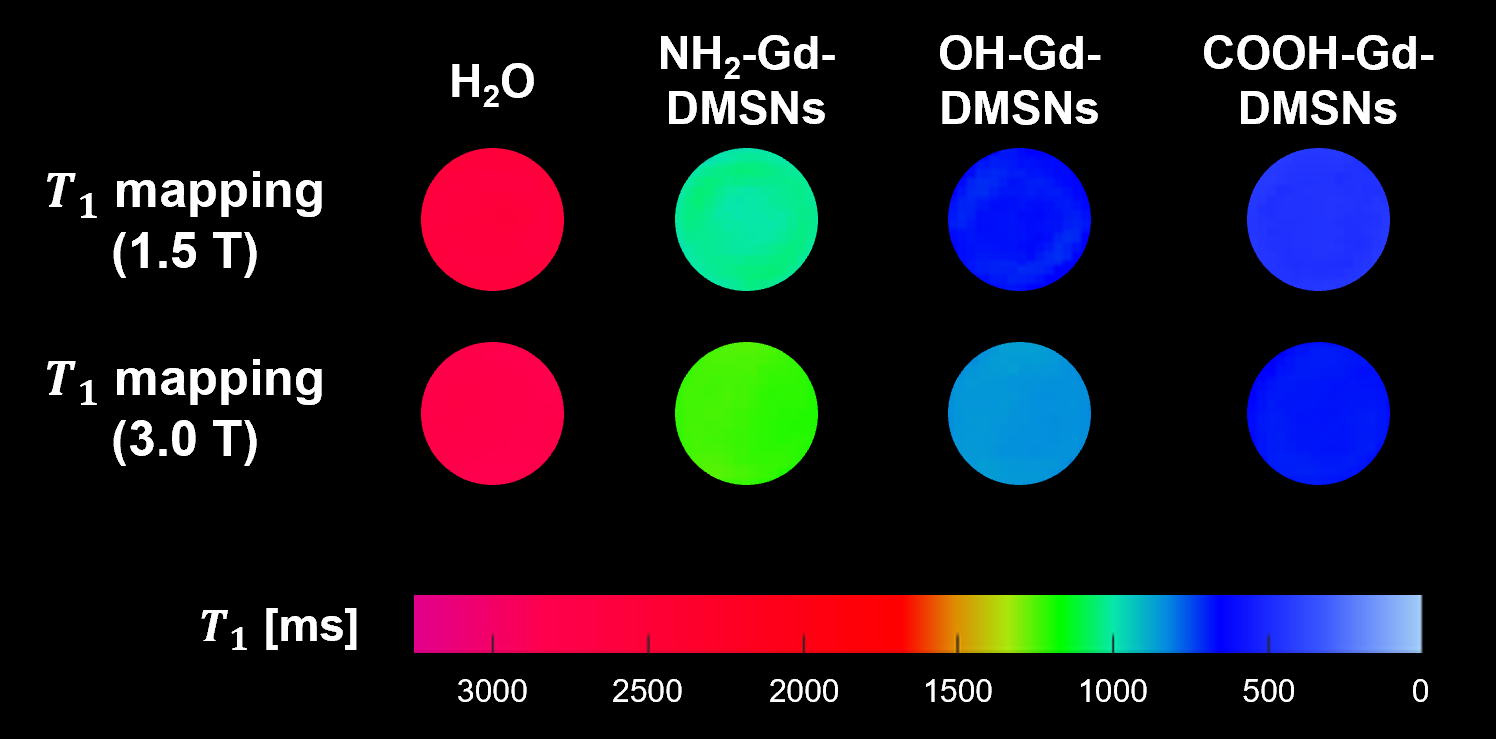
**

**Figure S28.** $T_{1}$ weighted MRI maps collected using clinical scanners for the NH_2_-Gd-DMSNs, OH-Gd-DMSNs and COOH-Gd-DMSNs recorded at both 1.5 T and 3 T. The corresponding $T_{1}$ times at 1.5 T are 2586 ± 29 ms for water, 1023 ± 14 ms for the NH_2_-Gd-DMSNs, 681 ± 12 ms for the OH-Gd-DMSNs and 466 ± 8 ms for the COOH-Gd-DMSNs. At 3 T the corresponding $T_{1}$ times are 2757 ± 35 ms for water, 1227 ± 23 ms for the NH_2_-Gd-DMSNs, 844 ± 9 ms for the OH-Gd-DMSNs and 686 ± 4 ms for the COOH-Gd-DMSNs.

**Crown ether modified MSNs results and discussion**


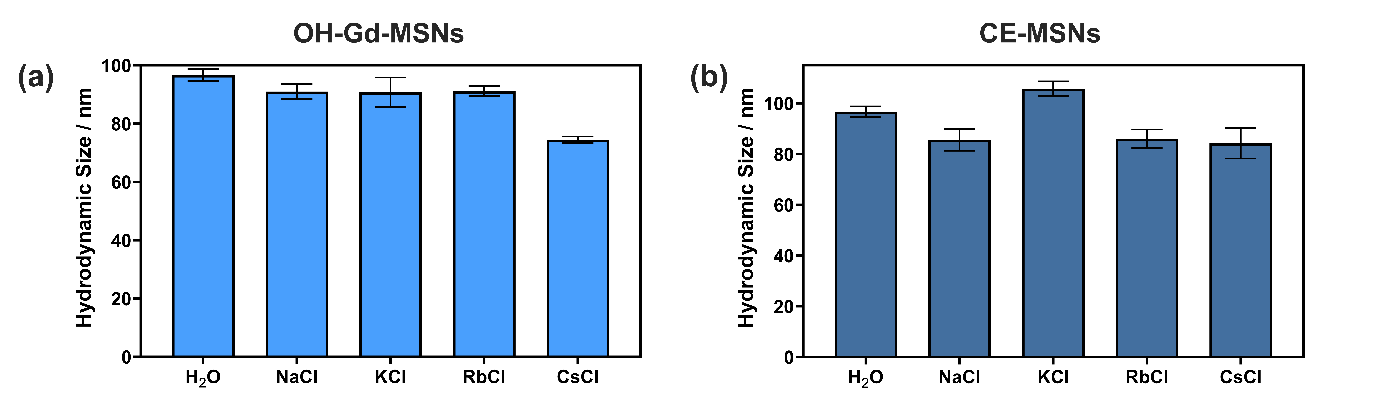


**Figure S29.** The hydrodynamic size, recorded by DLS, for a 1 mg mL^-1^ solution of (a) native OH-Gd-MSNs and (b) crown ether modified CE-MSNs, incubated in an aqueous 10 mM solution of the desired Group 1 chloride. These measurements show that all particles retain good colloidal stability on addition of the external salt.


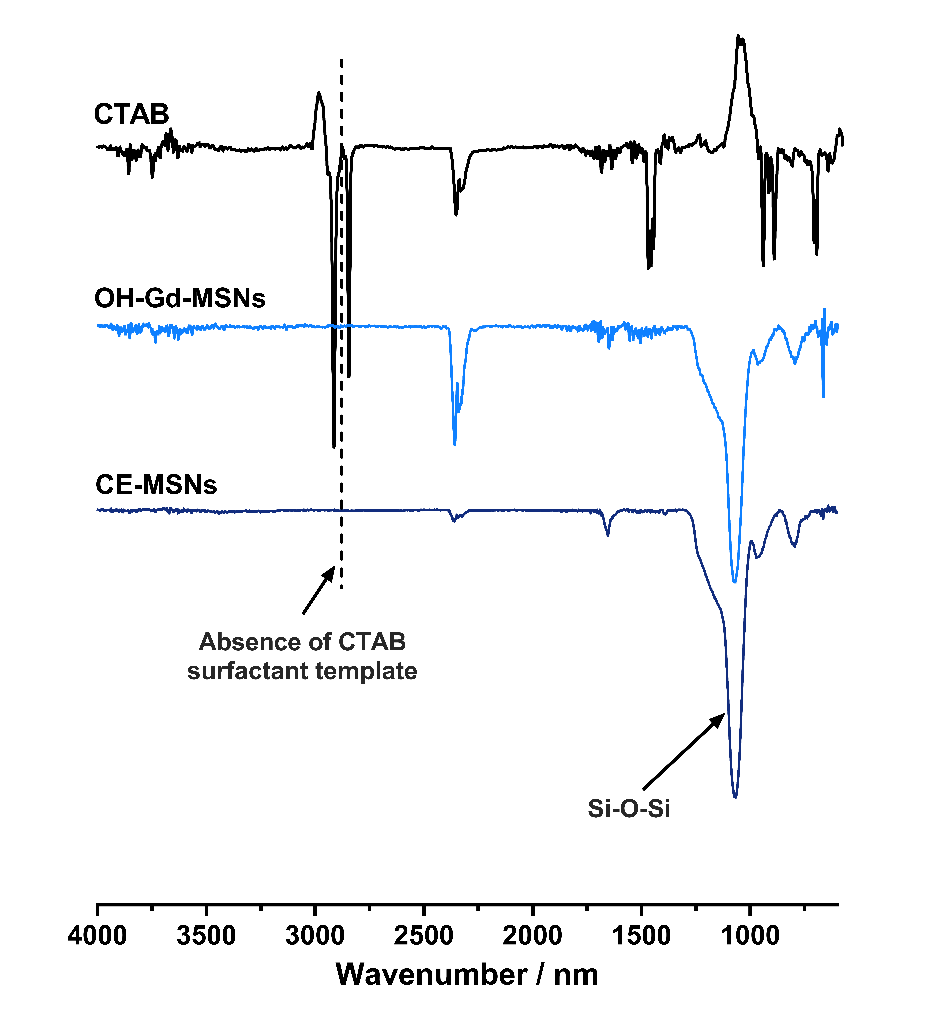


**Figure S30.** ATR-IR spectra for the CE-MSNs, highlighting the main architectural Si-O-Si stretch and absence of the templating CTAB surfactant.


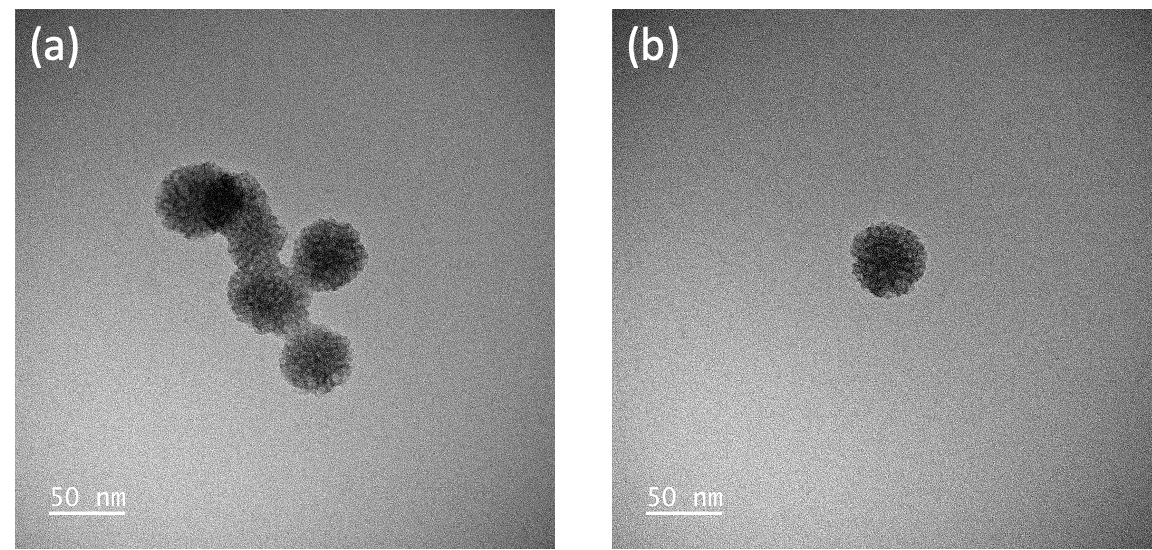


**Figure S31.** TEM images for the CE modified MSNs, with an associated particle diameter of 51 ± 7 nm.


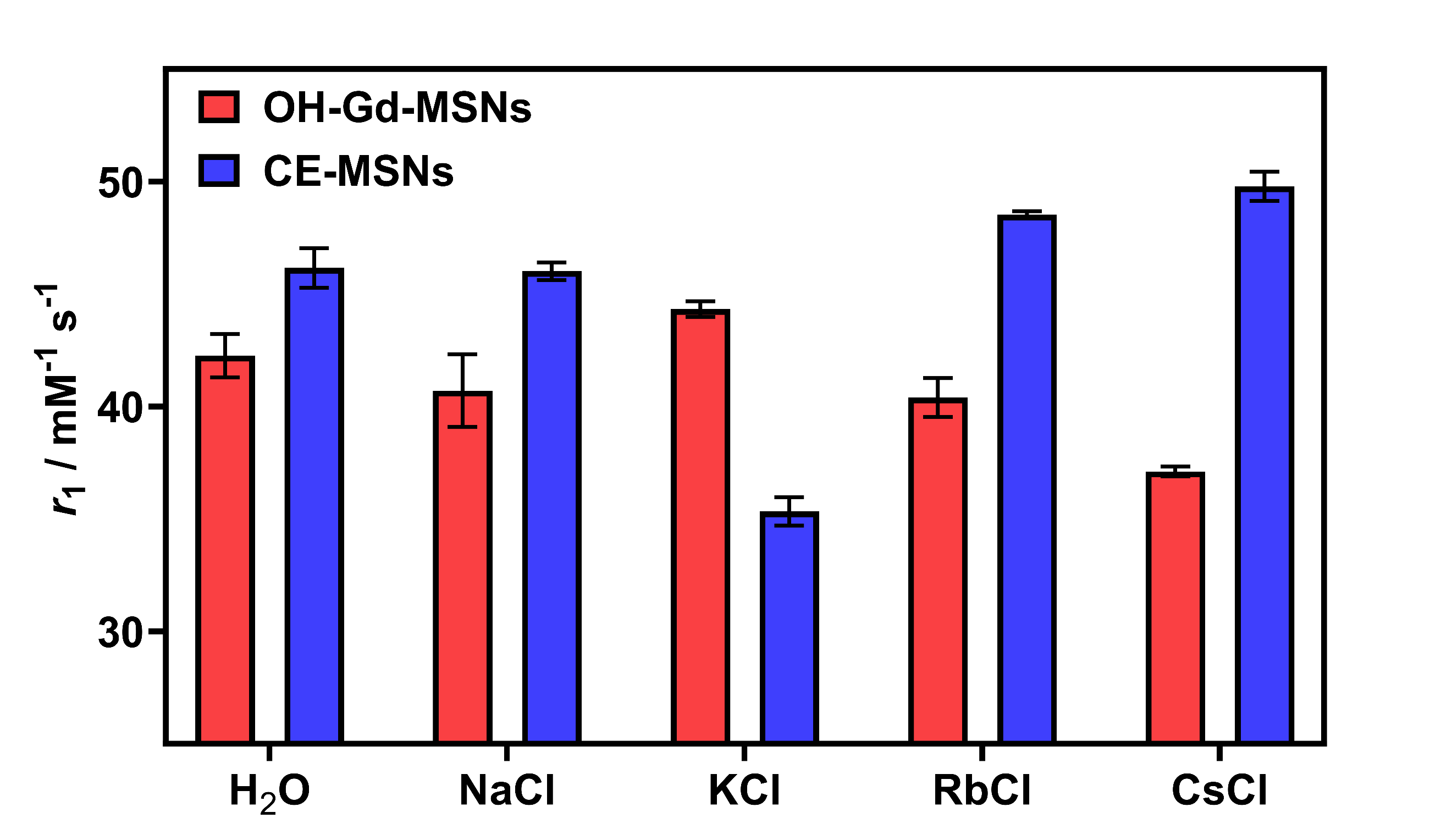


**Figure S32.** Relaxivity measurements of the native OH-Gd-MSNs and modified CE-MSNs for a 1 mg mL^-1^ solution of the MSNs in an aqueous 10 mM solution of the desired group 1 chloride.


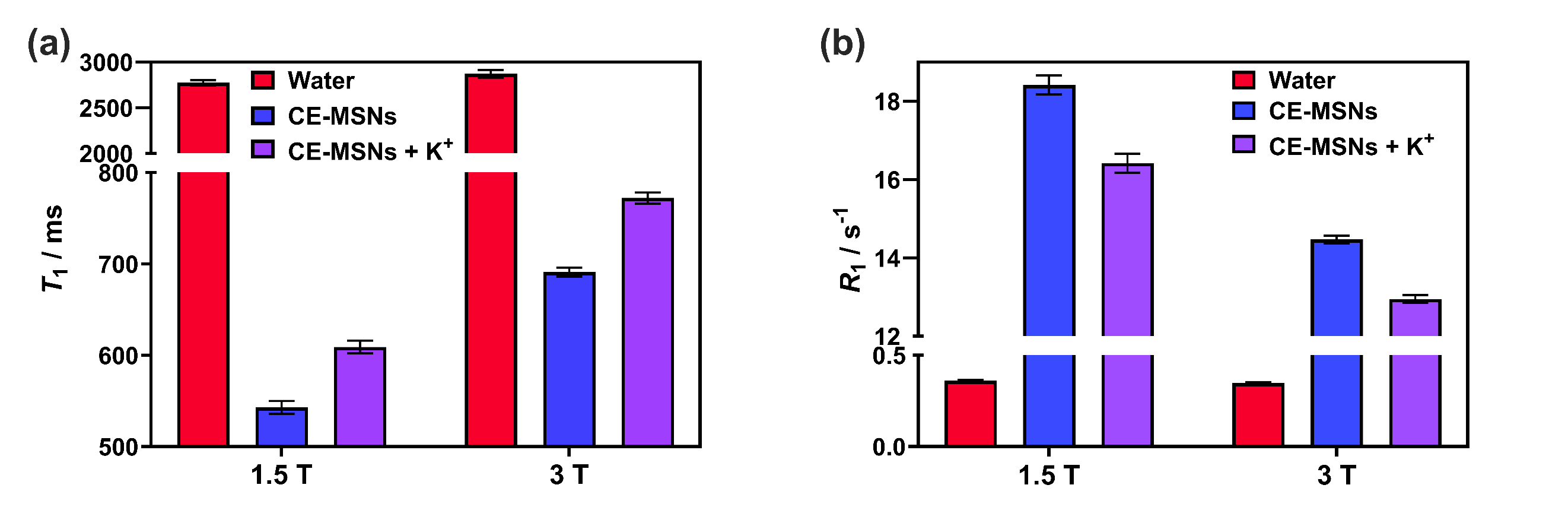


**Figure S33.** (a) MRI derived longitudinal relaxation times recorded at 1.5 T and 3 T for the crown ether modified MSNs in both the absence and presence of K^+^ (10 mM KCl). The corresponding $T_{1}$ times at 1.5 T are 2774 ± 29 ms for water, 543 ± 7 ms for the CE-MSNs in water and 609 ± 9 ms for the CE-MSNs in the presence of K^+^. The corresponding $T_{1}$ times at 3 T are 2873 ± 42 ms for water, 691 ± 5 ms for the CE-MSNs in water and 772 ± 6 ms for the CE-MSNs in the presence of K^+^. (b) The corresponding relaxation rate measurements for the crown ether modified MSNs in both the absence and presence of K^+^. The relaxation times and rates for water are included as a reference.

**

**

**Figure S34.** Δr_1_ values (initial CE-MSNs subtracting CE-MSNs with K^+^) as resolved by the MRI scanner at both 1.5 T and 3 T for CE-MSNs in the presence of 10 mM K^+^. All error bars correspond to ± 1 s d. of the measurement reported by the MRI scanner.

**References**

(1) Beeby, A.; M. Clarkson, I.; S. Dickins, R.; Faulkner, S.; Parker, D.; Royle, L.; S. de Sousa, A.; A. Gareth Williams, J.; Woods, M.; J. Chem. Soc., Perkin Trans. 2 1999, 3, 493–504.

(2) Piechnik, S. K.; Ferreira, V. M.; Dall'Armellina, E.; Cochlin, L. E.; Greiser, A.; Neubauer, S.; Robson, M. D.; J. Cardiovasc. Magn. Reson. 2010, 12, 69.

(3) Ong, W. Q.; Zhao, H.; Du, Z.; Yeh, J. Z. Y.; Ren, C.; Tan, L. Z. W.; Zhang, K.; Zeng, H.; Chem. Commun. 2011, 47 (22), 6416–6418.

(4) Kokan, Z.; Chmielewski, M. J.; J. Am. Chem. Soc. 2018, 140 (47), 16010–16014.

(5) Guo, Z.; Wu, L.; Wang, Y.; Zhu, Y.; Wan, G.; Li, R.; Zhang, Y.; Qian, D.; Wang, Y.; Zhou, X.; et al.; ACS Appl. Mater. Interfaces 2020, 12, 18823-18832.

(6) Caravan, P.; Farrar, C. T.; Frullano, L.; Uppal, R.; Contrast Media Mol. Imaging 2009, 4, 89-100.

(7) Caravan, P.; Ellison, J. J.; McMurry, T. J.; Lauffer, R. B.; Chem. Rev. 1999, 99, 2293-2352.

(8) Botta, M.; Eur. J. Inorg. Chem. 2000, 2000, 399-407.

(9) Aime, S.; Baranyai, Z.; Inorg. Chim. Acta 2022, 532, 120730.

(10) Powell, D. H.; Dhubhghaill, O. M. N.; Pubanz, D.; Helm, L.; Lebedev, Y. S.; Schlaepfer, W.; Merbach, A. E.; J. Am. Chem. Soc. 1996, 118, 9333-9346.

(11) Dunand, F. A.; Borel, A.; Merbach, A. E.; J. Am. Chem. Soc. 2002, 124, 710-716.

(12) Carniato, F.; Tei, L.; Cossi, M.; Marchese, L.; Botta, M.; Chem. Eur. J. 2010, 16, 10727-10734.

(13) Carniato, F.; Muñoz-Úbeda, M.; Tei, L.; Botta, M.; Dalton Trans. 2015, 44, 17927-17931.

(14) Carniato, F.; Tei, L.; Dastrù, W.; Marchese, L.; Botta, M.; Chem. Commun. 2009, 1246-1248.

(15) Lipari, G.; Szabo, A.; J. Am. Chem. Soc. 1982, 104, 4546-4559.

(16) Tei, L.; Gugliotta, G.; Baranyai, Z.; Botta, M.; Dalton Trans. 2009, 9712-9714.

(17) Rother, G.; Gautam, S.; Liu, T.; Cole, D. R.; Busch, A.; Stack, A. G.; J. Phys. Chem. C 2022, 126, 2885-2895.
